# Dissociation of Attentional State and Behavioral Outcome Using Local Field Potentials

**DOI:** 10.1523/ENEURO.0327-24.2024

**Published:** 2024-11-05

**Authors:** Surya S. Prakash, J. Patrick Mayo, Supratim Ray

**Affiliations:** ^1^Centre for Neuroscience, Indian Institute of Science, Bangalore 560012, India,; ^2^Department of Ophthalmology, University of Pittsburgh, Pittsburgh, Pennsylvania 15219

**Keywords:** gamma, high-gamma, macaque area V4, SSVEP, target detection, visual attention

## Abstract

Successful behavior depends on the attentional state and other factors related to decision-making, which may modulate neuronal activity differently. Here, we investigated whether attentional state and behavioral outcome (i.e., whether a target is detected or missed) are distinguishable using the power and phase of local field potential recorded bilaterally from area V4 of two male rhesus monkeys performing a cued visual attention task. To link each trial's outcome to pairwise measures of attention that are typically averaged across trials, we used several methods to obtain single-trial estimates of spike count correlation and phase consistency. Surprisingly, while attentional location was best discriminated using gamma and high-gamma power, behavioral outcome was best discriminated by alpha power and steady-state visually evoked potential. Power outperformed absolute phase in attentional/behavioral discriminability, although single-trial gamma phase consistency provided reasonably high attentional discriminability. Our results suggest a dissociation between the neuronal mechanisms that regulate attentional focus and behavioral outcome.

## Significance Statement

Targets appearing at the attended location are detected more accurately than those at the unattended location. However, attention may not be the only factor regulating the behavioral outcome. We investigated whether the effects of behavioral outcome and attentional state could be differentiated using the local field potentials recorded from macaque visual area V4. We used various methods to obtain single-trial estimates of trial-wise measures like correlations and phase consistency. Remarkably, we found that while attentional location was most effectively discerned through gamma and high-gamma power, behavioral outcomes were better distinguished by alpha power and steady-state visually evoked potentials. These results suggest distinct mechanisms underlying attention and behavioral outcome, thus emphasizing the roles of additional factors in modulating the behavioral outcome.

## Introduction

Attention facilitates behavior by selectively processing relevant sensory information (Carrasco, 2011). Attention improves behavioral performance by modulating neuronal signals such as spike rate (Motter, 1993; McAdams and Maunsell, 1999) and local field potential (LFP) oscillations in various frequency bands (Fries et al., 2001; Khayat et al., 2010; Prakash et al., 2021). The power in different frequency bands has often been used to decode the focus of attention (Esghaei and Daliri, 2014; Tremblay et al., 2015; De Sousa et al., 2021; Prakash et al., 2021). For example, we recently found that high-frequency LFP oscillations (>30 Hz) decode attention better than or on par with spikes when multiple electrodes are used (Prakash et al., 2021). This result contrasts with sensory (Kanth and Ray, 2020) and motor decoding (Hwang and Andersen, 2013) where multichannel spikes outperform multichannel LFP. To account for these differences in the mechanisms of cognitive versus sensorimotor processing, we proposed that the elevated performance of high-frequency LFPs is due to comparable spatial spreads of LFP and biophysical circuits that underlie attention (Prakash et al., 2022).

Our previous study (Prakash et al., 2021) probed a single aspect of behavior, the attentional state of the animal. Because there is no objective measure of where the monkey's attention was focused at a particular moment, the decoding may have been more related to which side the attentional cue was given and where the focus of attention was located on average. Specifically, we associated attention (as conventionally done) with cue-in [cue inside the receptive field (RF) of neurons being recorded] and cue-out conditions, which we termed “attention-in” and “attention-out” to be consistent with previous literature (Fries et al., 2001; Khayat et al., 2010). However, another relevant decoding variable that has been extensively studied is the behavioral outcome of a trial (“hit” for a detected target change versus “miss” for an undetected target change). Many studies have reported that the phase of low-frequency oscillations is informative for decoding behavioral outcome (Busch and VanRullen, 2010; Harris et al., 2018). However, whether decoding behavioral outcomes based on LFP features is as informative as decoding based on spikes is not known. This evaluation is critical for designing functional brain–computer interfaces and for potentially revealing the neural basis of attention and decision-making.

LFP phase consistency has been hypothesized to play a particularly important role in communication between neuronal ensembles and has been shown to be modulated by attention (Bosman et al., 2012; Grothe et al., 2012; Fries, 2015). For example, attention enhances the spike-LFP and LFP–LFP phase coherence in the gamma band in macaque visual area V4 (Fries et al., 2001; Buffalo et al., 2011; Prakash et al., 2021). Likewise, behavioral performance measured using reaction time also varies with the absolute LFP phase (Ni et al., 2016), spike-LFP phase coherence (Womelsdorf et al., 2006), and interareal LFP–LFP phase consistency (Rohenkohl et al., 2018) in the gamma band. Collectively, a substantial body of work suggests that the phase relationship between neuronal populations is systematically modulated by both attention and behavioral performance.

Because phase consistency measures are typically calculated across trials (but see Spyropoulos et al., 2024), they may fail to capture the well-established trial-by-trial changes in attentional fluctuations and behavioral performance that we seek to understand (Cohen and Maunsell, 2010, 2011; Ghosh and Maunsell, 2021; Roy et al., 2021). Thus, existing methods for calculating phase consistency may provide only a coarse estimate of the underlying dynamics of attention or behavioral state. This shortcoming is not limited to LFP measures. Conventional measures of spike count correlation between two neurons—which have been shown to reduce with attention (Cohen and Maunsell, 2009; Mitchell et al., 2009; Mayo and Maunsell, 2016)—suffer from the same limitation. To address these issues, we used several methods to compute pairwise metrics such as spike count correlation, LFP power correlation, and field–field coherence for single trials. We tested the ability of spikes, LFP power, LFP phase, and pairwise metrics in visual area V4 to independently decode attention and behavioral outcome of two rhesus macaques performing a standard change detection task.

## Materials and Methods

### Electrophysiological recordings

Data and experimental procedures used in this study are described in our previous studies (Mayo and Maunsell, 2016; Prakash et al., 2021). We outline the details briefly here. All animal procedures were approved by the Institutional Animal Care and Use Committee of Harvard Medical School. Two adult male rhesus monkeys (*Macaca mulatta*) were surgically implanted with a titanium headpost and scleral eye coil before training on the task. After training, each monkey was implanted with two 6 × 8 (48) microelectrode Utah arrays (Blackrock Microsystems), one in visual area V4 of each hemisphere. Electrodes were 1 mm long and 400 µm apart. The impedances of the electrodes were in the range of 0.2–1 MΩ at 1 KHz.

The raw signals from 96 channels were recorded using a 128-channel Cerebus Neural Signal Processor (Blackrock Microsystems). The single and multiunit spiking activities were obtained by filtering the raw signal between 250 Hz (fourth-order digital Butterworth filter) and 7.5 kHz (third-order analog Butterworth filter) and subsequently subjected to a voltage amplitude threshold and sorted offline using spike sorting software (Plexon). LFP signal was obtained by filtering the raw signal between 0.3 Hz (first-order analog Butterworth filter) and 2.5 kHz (fourth-order digital Butterworth filter), sampled at 2 kHz and digitized at 16-bit resolution. The possible aliasing caused by inadvertently setting the low-pass LFP filter cutoff at a frequency that was higher than the sampling frequency had a negligible effect on the LFP (Prakash et al., 2021).

### Behavioral task

**Figure 1.**
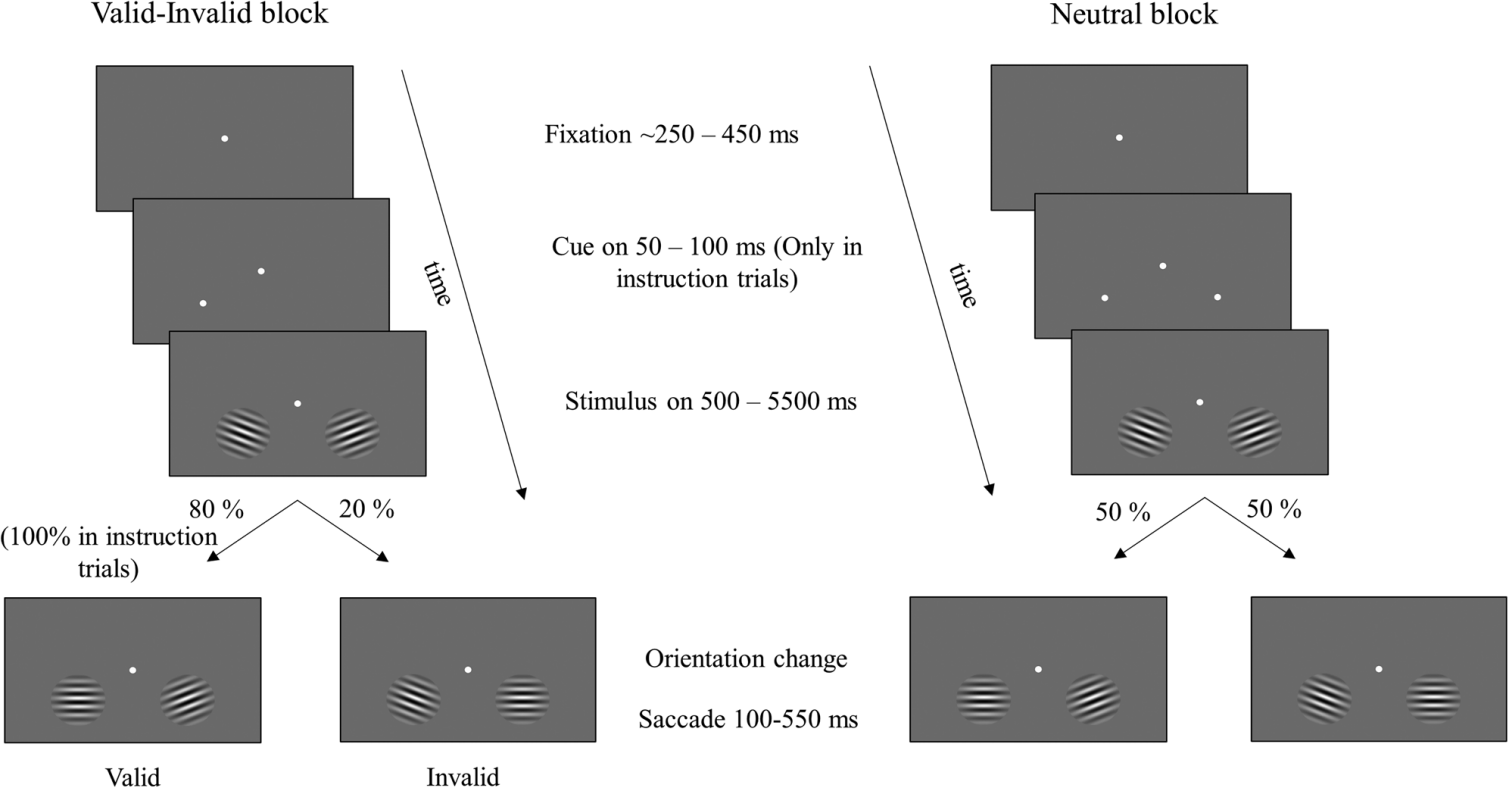
Schematic of orientation change detection task. Valid–invalid block (left panel), The task began with the appearance of a central fixation spot. Attention was cued in blocks using an initial set of “instruction trials” where the monkeys were explicitly cued to attend to a location (cued left in the depiction) by briefly flashing a white dot for 50–100 ms which indicated the target location with 100% certainty in the instruction trial and most likely location for rest of the block. Two Gabor stimuli counterphasing at 10 Hz appeared simultaneously and synchronously on the screen. At an unsignalled time between 500 and 5,500 ms after the stimulus onset, one of the Gabor stimuli changed its orientation (target), and the monkey was rewarded if it made a saccade to the location of orientation change between 100 and 550 ms after the target onset. The target appeared at the cued location with an 80% probability (valid cue) and at an uncued location with a 20% probability (invalid cue). Monkeys were rewarded for detecting the target at either of the locations. Once the monkeys performed four instruction trials correctly, then the explicit cue was no longer presented, and the target appeared probabilistically. Neutral block (right panel), Unlike the valid–invalid block, in the neutral block, the monkeys were cued by briefly flashing the white spot at both locations simultaneously during the instruction trials, and the target could occur at either of the two locations with 50% probability. The rest of the task structure is the same as that of the valid–invalid block.

Monkeys were trained to perform an orientation change detection task (Fig. 1). A trial began with the appearance of a small fixation spot at the center of the monitor, and the monkeys held their gaze within a 1.8° square window centered on the fixation spot throughout the trial. Two full contrast Gabor stimuli were presented simultaneously and synchronously on a CRT monitor (100 Hz frame rate, 1,024 × 768 pixels) over a uniform gray background, positioned 57 cm from the monkeys. Each Gabor was sinusoidally counterphased at 10 Hz, and its size, location, spatial frequency, and initial orientation were optimized for a randomly selected recording channel in each hemisphere during each session. At a random time chosen from an exponential distribution (mean, 3,000 ms; range, 500–5,500 ms) when the contrast of both the stimuli was at 0%, one of the stimuli changed its orientation. Monkeys were rewarded for making a saccade to the changed stimulus between 100 and 550 ms after the change. Eye position was sampled at 200 Hz using the scleral eye coil technique (Judge et al., 1980).

Monkeys were cued to attend to one (valid–invalid condition) or both stimuli (neutral condition) in blocks of ∼50 trials, each beginning with four correct instruction trials in which a small white spot appeared briefly for 50–100 ms indicating the location(s) of the change (target). In valid–invalid blocks, the target appeared at the cued location with 80% probability (valid cue) and 20% probability at the uncued location (invalid cue). In neutral blocks, the target appeared at either location with a 50% probability. Five percent of the total trials were catch trials in which no change occurred, and monkeys were rewarded for maintaining the fixation throughout the trial. Catch and instruction trials were not included in the analysis. Six different orientation change magnitudes were used for valid and neutral cue conditions, and only the second and third smallest orientation changes were used to probe attention in the invalid condition in each recording session.

### Data analysis

We analyzed the data from 25 sessions of two monkeys (Monkey A, 13 sessions; Monkey W, 12 sessions). We restricted our analysis to trials with either the second or third smallest orientation change in which the behavioral performance was close to 50% to obtain a comparable number of trials between hit and miss conditions. Note that the magnitude of orientation change can be different for different cue types and locations. In addition, we considered data for a particular cue condition of a given session only if there were >10 trials in each of their four conditions. Consequently, 22 out of 25 sessions met the criteria for valid and neutral cues but none for invalid cues. Hence, we only analyzed the data of valid and neutral cue conditions.

#### Mean matching of target-onset time distribution

**Figure 2.**
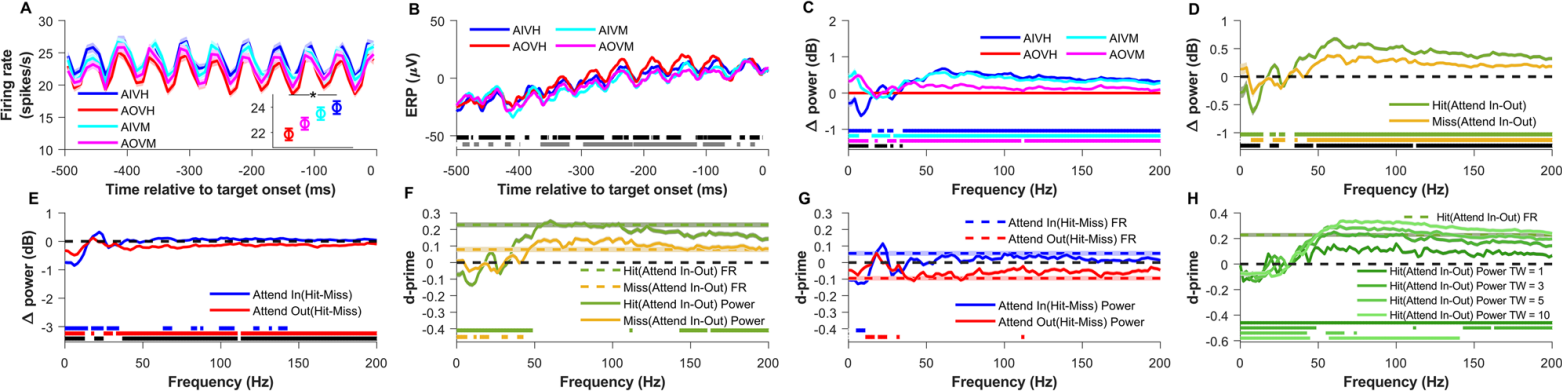
Comparison of firing rate (FR) and local field potential (LFP) power across validly cued attention and behavioral conditions for matched target onset time distributions. ***A***, Mean peristimulus time histogram (PSTH) relative to the target-onset time for the conditions in which attention was validly cued into or outside the receptive field of a neuron and the subject either detected (hit) or missed the target, namely, Attend-In Valid Hit (AIVH; blue), Attend-Out Valid Hit (AOVH; red), Attend-in Valid Miss (AIVM; cyan), and Attend-Out Valid Miss (AOVM; magenta). Inset shows the mean firing rate over the same time period as PSTH for the four conditions. The mean is first taken across 643 electrodes recorded across 21 sessions in two monkeys and then averaged across 50 bootstrap iterations of target-onset time-matched trial selection. The shaded lines and error bars indicate the bootstrap mean of SEM across the 643 electrodes. Only sessions in which at least 10 stimulus repeats were available for every condition were used for analysis. The asterisk in the inset indicates that the mean firing rate of AIVH is significantly higher than AOVH (Wilcoxon rank-sum test; Bonferroni corrected *p* < 0.05). ***B***, Mean event-related potential (ERP) for the four conditions. The horizontal black patches at the bottom indicate the time values during which the ERP of AIVH and AIVM were significantly different (Wilcoxon rank-sum test; Benjamini–Hochberg FDR controlled *p* < 0.05), and the gray patches indicate the time values during which ERP of AOVH and AOVM were significantly different (Wilcoxon rank-sum test; Benjamini–Hochberg FDR controlled *p* < 0.05). ***C***, Mean change in power spectral density (PSD) in decibels for all the validly cued conditions relative to the Attend-out Valid Hit (AOVH) condition. The horizontal blue, cyan, and magenta patches at the bottom indicate the frequencies at which the change in PSD relative to the AOVH condition is significantly different from zero of the conditions represented by their corresponding color (Wilcoxon signed-rank test, Benjamini–Yekutieli FDR controlled *p* < 0.05 under unknown dependency). The black horizontal patches indicate the frequencies at which the change in PSD of AIVH (blue trace) and AIVM (cyan trace) are significantly different from each other (Wilcoxon rank-sum test, Benjamini–Yekutieli FDR controlled *p* < 0.05 under unknown dependency). ***D***, Mean change in LFP power spectral density (PSD) in decibels between attend-in and attend-out conditions for hit (AIVH and AOVH; green) and miss (AIVM and AOVM; yellow) conditions. The horizontal dashed black line marks the zero of the *y*-axis. The horizontal green and yellow patches at the bottom indicate the frequencies at which the change in PSD between attention conditions are significantly greater than zero for hits and miss conditions, respectively (Wilcoxon signed-rank test, Benjamini–Yekutieli FDR controlled *p* < 0.05 under unknown dependency). The horizontal black patch at the bottom indicates the frequencies at which the change in PSD between attention conditions for hit and miss conditions are significantly different from each other (Wilcoxon rank-sum test, Benjamini–Yekutieli FDR controlled *p* < 0.05 under unknown dependency). ***E***, Mean change in power spectral density between hit and miss conditions for Attend-In (AIVH and AIVM; blue) and Attend-Out (AOVH and AOVM; red) conditions. The horizontal dashed black line marks the zero of the *y*-axis. The horizontal blue and red patches at the bottom indicate the frequencies at which the change in PSD between behavioral conditions are significantly greater than zero for attend-in and attend-out conditions, respectively (Wilcoxon signed-rank test, Benjamini–Yekutieli FDR controlled *p* < 0.05 under unknown dependency). The horizontal black patch at the bottom indicates the frequencies at which the change in PSD between behavioral conditions for attend-in and attend-out condition are significantly different from each other (Wilcoxon rank-sum test, Benjamini–Yekutieli FDR controlled *p* < 0.05 under unknown dependency). ***F***, Mean *d*′ (the ratio of the mean difference and pooled standard deviation of two conditions) of LFP power (solid lines) and firing rate (dashed color lines) between attend-in and attend-out conditions for hit (green) and miss (yellow) conditions. The horizontal dashed black line marks the zero of the *y*-axis. The horizontal green and yellow patches at the bottom indicate the frequencies at which the *d*′ of LFP power is significantly different from the *d*′ of firing rate for hit and miss conditions, respectively (Wilcoxon rank-sum test, Benjamini–Yekutieli FDR controlled *p* < 0.05 under unknown dependency). Negative *d*′ values were multiplied by −1 before performing the significance test because we were interested in the differences in magnitude of *d*′. ***G***, Mean *d*′ of LFP power (solid lines) and firing rate (dashed color lines) between hit and miss conditions for Attend-In (blue) and Attend-out (red) conditions. The horizontal dashed black line marks the zero of the *y*-axis. The horizontal blue and red patches at the bottom indicate the frequencies at which the *d*′ of LFP power is significantly different from the *d*′ of firing rate for attend-in and attend-out conditions, respectively (Wilcoxon rank-sum test, Benjamini–Yekutieli FDR controlled *p* < 0.05 under unknown dependency). Negative *d*′ values were multiplied by −1 before performing the significance test as in ***F***. ***H***, Mean *d*′ of LFP power between attend-in and attend-out conditions for hit condition where LFP power is estimated by multitaper method using time–frequency bandwidth product (TW) of 1 (darkest green), 3 (dark green), 5 (light green), and 10 (lightest green). The dashed green line indicates the *d*′ of firing rate between the same conditions. The horizontal dashed black line marks the zero of the *y*-axis. The horizontal colored patches at the bottom indicate the frequencies at which the *d*′ of LFP power estimated using different TW is significantly different from the *d*′ of firing rate (Wilcoxon rank-sum test, Benjamini–Yekutieli FDR controlled *p* < 0.05 under unknown dependency). Negative *d*′ values were multiplied by −1 before performing the significance test as in ***G*** and ***F***. A similar comparison of the firing rate and LFP power for valid conditions before matching the target-onset time distribution and for neutral conditions after matching the distributions are shown in Extended Data Figure 2-1.

We matched the target-onset time distribution of hit and miss trials of each attention condition to remove the artifactual drift introduced due to the difference in the number of early target-onset (<750 ms) trials. We sorted the hit and miss trials separately based on the target-onset times (500–5,500 ms) and divided them into bins of 250 ms and randomly chose an equal number of trials from both conditions in each bin. We performed the analysis (Fig. 2 onwards) on the mean-matched data and computed all the neural measures. The process was repeated 50 times and then averaged across iterations.

10.1523/ENEURO.0327-24.2024.f2-1 Figure 2-1 **Comparison of firing rate (FR) and local field potential (LFP) power across (1) validly cued attention and behavioral conditions for non-matched target onset time distributions and (2) neutrally cued attention and behavioral conditions for matched target onset time distributions.** (A) – (G) Same as Figure 2 (A) – (G) but for the case where the target onset time distributions of hit and miss conditions were not matched. Here the mean is taken across 677 electrodes recorded across 22 sessions in two monkeys. Shaded lines and error bars (not visible for most traces) indicate the s.e.m across the 677 electrodes. (H) Frequency distribution of target onset time for the four validly cued conditions of all the sessions. The number in the brackets indicate total number of trials in the respective conditions. (I) Mean peri-stimulus time histogram (PSTH) relative to the target onset time for the neutrally cued conditions in which attention was cued to both visual hemifields simultaneously and target could appear at either of the location with 50% probability. Unlike the valid cue condition where the conditions were divided based on attention location, here the conditions are divided based on where the target eventually appeared, namely Target-In Neutral Hit (TINH; blue), Target-Out Neutral Hit (TONH; red), Target-In Neutral Miss (TINM; cyan), Target-Out Neutral Miss (TONM; magenta). Inset shows the mean firing rate over the same time period as PSTH for the four conditions. Mean is first taken across 659 electrodes recorded across 21 sessions in two monkeys and then averaged across 50 bootstrap iterations. Shaded lines and error bars indicate the bootstrap mean of s.e.m across the 659 electrodes. (J) Mean ERP for the four conditions described in A. (K) Mean change in power spectral density for all the neutrally cued conditions relative to Target-Out Neutral Hit (TONH) condition. (L) Mean change in LFP power spectral density in decibels between Target-In and Target-Out conditions for hit (TINH and TONH; green) and miss (TINM and TONM; yellow). The horizontal dashed black line marks the zero of the y-axis. (M) Mean change in LFP power spectral density between hit and miss condition for neutrally cued Target-In (TINH and TINM; blue) and Target-Out (TONH and TONM; red) conditions. The horizontal dashed black line indicates the zero of the y-axis. (N) Mean d-prime of LFP power (solid lines) and firing rate (dashed color lines) between neutrally cued attend-in and attend-out conditions for hit (green) and miss (yellow) conditions. The horizontal dashed black line marks the zero of the y-axis. (O) Mean d-prime of LFP power (solid lines) and firing rate (dashed color lines) between hit and miss condition for neutrally cued Target-In (blue) and Target-out (red) conditions. The horizontal dashed black line indicates the zero of the y-axis. (P) Mean d-prime of LFP power between Target-In and Target-Out conditions for hit condition where LFP power is estimated by Multitaper method using time-frequency bandwidth product (TW) of 1 (darkest green), 3 (dark green), 5 (light green) and 10 (lightest green). The dashed green line indicates the d-prime of firing rate between the same conditions. The horizontal dashed black line marks the zero of the y-axis. Asterisks and horizontal color patches at the bottom of each panel indicate the significance level like in Figure 2. Download Figure 2-1, TIF file.

#### Spiking and LFP analysis

We analyzed the spikes and LFP data between 500 and 0 ms before the target (orientation change) onset to obtain the following measures.

#### Single-electrode measures

#### Trial-wise measures

##### Peristimulus time histogram

Prestimulus time histogram (PSTH) was obtained by counting spikes in nonoverlapping 10 ms bins for all the trials and converting them into firing rates which were then averaged across trials.

##### Evoked response potential

Evoked response potential (ERP) is obtained by trial averaging the LFP signal in the analysis period.

##### Single-electrode pairwise phase consistency

Traditionally, pairwise phase consistency (PPC) quantifies the consistency of phase difference between two signals, like spike and LFP from the same electrode or spike-LFP or LFP–LFP from two different electrodes (Vinck et al., 2010). In single-electrode PPC, we simply used the absolute phase rather than the phase difference. We estimated five phase values using the multitaper method with five Slepian tapers (Jarvis and Mitra, 2001) using the Chronux toolbox (RRID:SCR_005547; Bokil et al., 2010) in MATLAB (RRID:SCR_001622). Then, we calculated the PPC for the phase estimate of each taper and averaged the PPC values across tapers to obtain the PPC value for each electrode.

#### Single-trial measures

##### Power spectral density

We estimated the power of the LFP signal in the 0–200 Hz frequency range for each trial with 2 Hz resolution using the multitaper method with five Slepian tapers.

##### Phase

Phase was estimated by taking the Fourier transform of the LFP signal. To account for the circular nature of the phase data, we used the sine component of the phase angle as the measure.

#### Electrode pairwise measure

##### Trial-wise measure

*Firing rate (FR)/LFP power correlation.* Pearson’s correlation between the firing rates or LFP powers of pairs of electrodes was computed across trials.

*LFP–LFP PPC*. The phase relationship between the LFP signals of pairs of electrodes was quantified using PPC, an unbiased estimate of square of the phase lock value measuring the consistency of the phase difference of LFP signals between two electrodes across pairs of trials (Vinck et al., 2010). We obtained five phase angles at each frequency for each electrode using the multitaper method with five Slepian tapers and then computed the PPC of taper averaged phase difference between two electrodes across pairs of trials.

##### Single-trial measure

Traditionally, electrode pairwise measures like correlation and PPC are measured across trials. However, we needed a single-trial measure of these pairwise measures to predict the attention and behavioral state of the monkeys on each trial. Hence, we developed the following two methods to estimate the correlation and phase consistency of a single trial.

1. Binning: We divided spiking and LFP data of 500 ms length into 10 nonoverlapping segments of 50 ms each. We computed firing rate and LFP power using Fourier transform in each segment resulting in 10 estimates of firing rate and LFP power with a frequency resolution of 20 Hz. Correlation and phase consistency were computed across these ten estimates. Since this bin-wise correlation captures the fluctuations over time, factors like adaptation where the activity of the neurons reduces over time can also give rise to spurious correlation. To remove the effects of such factors, we shuffle corrected the correlation values (Smith and Kohn, 2008). We calculated bin-wise correlation values for a given trial with every other nonsimultaneous trial and averaged it across all the trial pairs. We then subtracted the trial pair averaged correlation from the original bin-wise correlation of the corresponding trial.
2. Multitaper: Multiple independent estimates of LFP power and phase for a trial can also be obtained using the multitaper method, where a single-trial LFP data is multiplied by several orthogonal tapers and then Fourier transformed to obtain several estimates of power (by squaring the amplitude) and phase (Fig. 4). We used nine tapers to obtain nine independent estimates of power and phase for a trial and computed correlation and phase consistency across them.

In addition to the above two methods, we also calculated single-trial estimates of LFP power correlation and pairwise phase consistency using the Hilbert transform method (Spyropoulos et al., 2024). We bandpass filtered the LFP signal within the analysis period using a fourth-order Butterworth filter of center frequency starting from 6 Hz with a frequency bandwidth of 10 Hz (i.e., center frequency ±5 Hz) moving in step of 2 Hz up to 200 Hz. The LFP signal filtered around each center frequency was Hilbert transformed to obtain instantaneous amplitude and phase values at each of the 1,000 timepoints within the 500 ms analysis window. Instantaneous power was calculated by squaring the amplitude. Finally, correlation in power and phase consistency were calculated across timepoints for every trial, and correlation values were shuffle corrected.

#### Discriminability using *d*′

We were interested in quantifying the separation in the distributions of the responses for two classes (attend-in vs attend-out or hit vs miss). Such discriminability is typically quantified using *d*′, which quantifies the difference in the means of the two distributions normalized by their average standard deviation. Here, we used pooled standard deviation to account for an unequal number of trials between conditions. The advantage of this measure is that no explicit threshold needs to be assumed for classification. Another equivalent metric is the area under the receiver operating characteristic curve [area under the curve (AUC)], which is related to *d*′ for normally distributed classes by AUC = 0.5 + erf (*d*′ / 2) / 2, where erf is the error function. Therefore, *d*′ values of 0.1, 0.2, and 0.3 (which is about the maximum we obtained in our data) correspond to AUC values of 0.52, 0.55, and 0.58, respectively. We used *d*′ values for discriminability due to their ease of computation and interpretation.

The *d*′ metric is given by the formula:

$$d^{\prime }=\frac{\mu_{1}-\mu_{2}}{\sqrt{\frac{(n_{1}-1)\sigma_{1}^{2}+(n_{2}-1)\sigma_{2}^{2}}{n_{1}+n_{2}-2}}},$$

where $\mu_{1}$is the mean across trials of attend-in/target-in or hit condition, $\mu_{2}$ is the mean across trials of attend-out/target-out or miss condition, $\sigma_{1}^{2}$ is the variance across trials of attend-in/target-in or hit condition, $\sigma_{2}^{2}$ is the variance across trials of attend-out/target-out or miss condition, $n_{1}$ is the number of trials in attend-in/target-in or hit condition, and $n_{2}$ is the number of trials in attend-out/target-out or miss condition

#### Discriminability of traditional frequency bands

We compared the discriminability of the neural measures in the following standard frequency bands: alpha (8–12 Hz), gamma (40–80 Hz), high-gamma (120–200 Hz), and steady-state visually evoked potential (SSVEP; 18–22 Hz) frequency. We chose SSVEP frequency over a band of frequency rather than a single-frequency point because of frequency smoothing caused by using five tapers. We obtained the power and PPC in each band, by averaging them over the frequencies of their respective bands and then computed their *d*′. We excluded the harmonics of SSVEP frequency (multiples of 20) in the gamma and high-gamma bands. However, for the binning method where the frequency resolution was 20 Hz, we excluded only 60 Hz. We computed the correlations of the frequency-averaged power.

We could not analyze the behavioral discriminability (hits vs miss) at the population level by combining the activity of all the electrodes like we did previously (Prakash et al., 2021) for attention discriminability using linear discriminant analysis (Fisher, 1936) because there were an insufficient number of miss trials. The invertibility of the covariance matrix (required to obtain the weight vector) is guaranteed when the sample size is greater than the dimension (Li et al., 2006). But in our dataset, the number of missed trials [∼20 trials (samples) per session on average after matching the target-onset times] was lesser than the number of electrodes [∼30 electrodes (dimension) per session on average]. Although this limitation can be overcome by regularization (Guo et al., 2007), it underestimates the discriminability (Prakash et al., 2021).

### Statistical analysis

We performed a Wilcoxon signed-rank test to compare whether the difference between attention and behavioral conditions for each neural measure was different from zero. We used the Wilcoxon rank-sum test to compare the attention and behavioral differences across neural measures and discriminability (*d*′) of different neural measures. Negative *d*′ values were converted to positive values by multiplying with −1 before performing the significance test since we were comparing only the magnitude. All the *p*-values are reported after correcting for the multiple comparisons using Bonferroni’s correction or Benjamini–Hochberg procedure controlling for false discovery rate (FDR) for independent statistical tests and Benjamini–Yekutieli procedure for dependent statistical tests under unknown dependency. Multiple comparison test was done using the Multiple Testing Toolbox (Martínez-Cagigal, 2024) in MATLAB. We performed the statistical tests on the bootstrap averaged measures across the electrodes.

### Data and code accessibility

The data and code used in the paper are freely available online in the following GitHub repository: https://github.com/surya0021/MayoProject2.

## Results

We recorded spike activity and local field potentials (LFPs) using microelectrode arrays implanted in both hemispheres of area V4 in two monkeys. Monkeys performed an attention task in which two Gabor stimuli that counterphased at 10 Hz were presented on a screen, and the monkeys were cued to either attend to one or both stimulus locations (see Materials and Methods for details) in separate blocks of trials (Fig. 1). In trials where one location was cued, the target (change in the orientation of the Gabor) occurred on the cued side in 80% of trials (validly cued) and on the uncued side in 20% of trials (invalidly cued; Fig. 1, left panel). In addition, there were trials where both sides were simultaneously cued (neutral cue), and the target could appear on either side with equal probability (Fig. 1, right panel).

Trials were categorized into different conditions based on cue/target locations (attend/target inside or outside the RF), cue types (valid, neutral, or invalid), and behavioral outcomes (hit or miss). Invalidly cued trials were not analyzed due to the insufficient number of trials (see Materials and Methods, Data analysis for more details). Since the monkeys were cued to attend to both locations in neutral blocks, we categorized those trials based on the target location instead of the cue location. This resulted in the following eight conditions (four each for valid and neutral cueing): Attend-In Valid Hit (AIVH), Attend-Out Valid Hit (AOVH), Attend-In Valid Miss (AIVM), Attend-Out Valid Miss (AOVM), Target-In Neutral Hit (TINH), Target-Out Neutral Hit (TONH), Target-In Neutral Miss (TINM), and Target-Out Neutral Miss (TONM)

### Effect of attention and behavior on firing rate and LFP power

We found that differences in target-onset times for hits versus miss conditions had a strong effect on our results, especially at low frequencies (as discussed below in more detail). Therefore, we analyzed the data after matching the target-onset time distributions of the hit and miss trials of the respective attention conditions (see Materials and Methods for details). Extended Data Figure 2-1*A–G* shows the same analyses as Figure 2*A–G* but before matching the target-onset time distribution (Extended Data Fig. 2-1*H*).

Figure 2*A* shows the average peristimulus time histogram (PSTH) across 643 electrodes across 21 sessions for the four validly cued conditions during 500–0 ms before the target onset (see Materials and Methods for details). Neurons fired at both the positive and negative phases of the counterphasing stimulus to produce a strong oscillatory signal at 20 Hz (De Valois et al., 1982). The average firing rates over this 500 ms interval are shown in the inset. The firing rate was higher when the monkeys attended and detected (hit) the target presented inside the RF (Attend-In Valid Hit condition; blue) than when they attended and detected the target outside the RF (Attend-Out Valid Hit condition; red; Bonferroni corrected *p* = 0.01; Wilcoxon rank-sum test). This recapitulates the classic effect of visual attention on the firing rates of cortical neurons, whereby the responses to attended stimuli in the RF are enhanced, on average, relative to unattended stimuli (McAdams and Maunsell, 1999). The conditions when the monkeys missed the target (Attend-Out Valid Miss, magenta, and Attend-In Valid Miss, cyan) produced a response that was intermediate between the two hit conditions since attention is expected to be on the uncued side leading to the miss.

Event-related potential (ERP), obtained by averaging the LFP traces for different conditions, showed similar trends across four conditions (Fig. 2*B*). However, these traces showed a pronounced difference between the hit and miss conditions when the target-onset time distributions were not matched (Extended Data Fig. 2-1*B*). These differences could be largely explained based on differences in target-onset times for hits versus miss conditions because monkeys tended to miss the targets that appeared earlier after stimulus onset than later (Extended Data Fig. 2-1*H*). Stimulus onset produced a transient elevation in firing rate and a dip in the ERP which reached a steady-state value over several hundred milliseconds [Prakash et al. (2021), their Fig. 1*A,B*]. Thus, because our analysis window was from 500 to 0 ms before the target onset (which could appear between 500 and 5,500 ms of stimulus onset), earlier target-onset times captured the transient activity for the trials in which the target appeared between 500 and 750 ms after the stimulus onset.

We next examined the effect of attention and behavioral outcomes on LFP power. Figure 2*C* shows the change in power of each condition from the Attend-Out Valid Hit condition. Attention suppressed the power in lower frequencies (≤14 Hz; blue trace; Benjamini–Yekutieli FDR controlled *p* < 0.05; Wilcoxon sign-rank test) and increased power in the gamma and high-gamma range (>36 Hz; Benjamini–Yekutieli FDR controlled *p* < 4.4 × 10^−16^; Wilcoxon sign-rank test) as well as the steady-state visually evoked potential (SSVEP) frequency of 20 Hz (Benjamini–Yekutieli FDR controlled *p* = 0.001; Wilcoxon sign-rank test). The effect of behavioral outcome (hit vs miss) was observed when attention was outside the RF and the monkey missed the target (magenta trace; Attend-Out Valid Miss vs Attend-Out Valid Hit) or by comparing the corresponding conditions for the Attend-In condition (i.e., blue vs cyan traces; Attend-In Valid Hit vs Attend-In Valid Miss). Upon visual inspection, low frequencies captured differences in activity related to behavioral outcome, while frequencies closer to gamma distinguished attentional state.

To better dissociate the effects of attentional location and behavioral outcome and examine how the effect of one is influenced by the other, we compared the power spectral densities separately by taking the difference between the attention conditions (attend-in vs attend-out) for the two behavioral outcomes: hit (Fig. 2*D*, green trace; Fig. 2*C*, same as the blue trace) and miss (Fig. 2*D*, yellow trace; Fig. 2*C*, cyan minus magenta traces). Attention-related suppression of low-frequency power (8–14 Hz; Benjamini–Yekutieli FDR controlled *p* < 0.01; Wilcoxon sign-rank test) and enhancement of the high-frequency power (>44 Hz; Benjamini–Yekutieli FDR controlled *p* < 0.05; Wilcoxon sign-rank test) was also observed for the miss condition, albeit the effect was weaker than the hit condition (Fig. 2*D*, compare green vs yellow traces). Similarly, for behavioral effects, we took the difference between the behavioral outcomes (hit − miss) for the two attention conditions: attend-in (Fig. 2*E*, blue trace; Fig. 2*C*, blue minus cyan traces) and attend-out (Fig. 2*E*, red trace; Fig. 2*C*, same as the negative of the magenta trace). For the behavioral comparison (Fig. 2*E*), the power was different mainly in the low-frequency range for both attend-in and attend-out cases, whereas high-frequency power showed only minor differences mainly for the attend-out condition.

Overall, our results show only a modest effect of behavior on LFP data that is mainly in the low-frequency range. But this is largely due to the target-onset matching procedure described above. The difference in power in the very low-frequency range for the missed condition relative to the hit condition was much larger for the nonmatched case (Extended Data Fig. 2-1*E*) compared with the matched case (Fig. 2*E*). For example, the change in 4 Hz power of attend-in valid miss (AIVM) relative to attend-in valid hit (AIVH) reduced by more than threefold in distribution matched (0.75 dB) compared with nonmatched case (2.4 dB; Fig. 2*E*, Extended Data Fig. 2-1*E*). This shows that the pretarget low-frequency power can spuriously dominate and lead to an overestimation of its behavioral decoding performance if the differences in the distribution of target-onset times between the conditions are not accounted for. For all the analyses, we therefore used trials only after matching target-onset time distributions.

### Attentional and behavioral discriminability using firing rate and LFP power

Since the main aim of the study was to compare the ability of different neural measures (such as spiking activity, LFP power, and LFP phase) to discriminate attentional conditions and behavioral outcome, we converted the LFP power and spike responses to *d*′ values to compare the discriminability across neural measures (Fig. 2*F,G*; see Materials and Methods for details). Attention discriminability using firing rates (indicated by dashed lines in Fig. 2*F*) was higher for hit condition (green dotted line) than miss (yellow dotted line), reflecting a larger separation in the means for the hits compared with miss cases (as in Fig. 2*A* inset). Attention discriminability with LFP power was highest in the gamma band (40–80 Hz), comparable to that of firing rate, and decreased slowly at higher frequencies (Fig. 2*F*, solid green and yellow traces). Lower frequencies (<30 Hz) had negative *d*′ (i.e., the attend-in condition had lower power than attend-out as shown in Fig. 2*D*), but the magnitude of the discriminability was lower than that of the firing rate. On the other hand, very low-frequency (<14 Hz) power was best suited for discriminating the behavior (Fig. 2*G*) since it had the highest magnitude of discriminability, reflecting the changes in power observed in Figure 2*E*.

To summarize, two salient results so far are as follows: (1) *d*′ values for LFP power for attentional discriminability (Attend-In vs out; Fig. 2*F*) for hit (green trace) and miss condition (yellow trace) were highest in the gamma band, where it was comparable to *d*′ of firing rate, and decreased in the high-gamma range. It was negative at low frequencies, although the magnitude was less than the gamma range. (2) For behavioral discriminability (Fig. 2*G*) for attend-in condition (blue solid trace), *d*′ values were negative at low frequencies, became positive at SSVEP frequency and in high-gamma range, comparable to the discriminability using firing rate (blue dashed line). The highest *d*′ was observed for the SSVEP frequency. The effect flipped for the attend-out condition in the high-gamma range (red solid trace).

**Figure 3.**
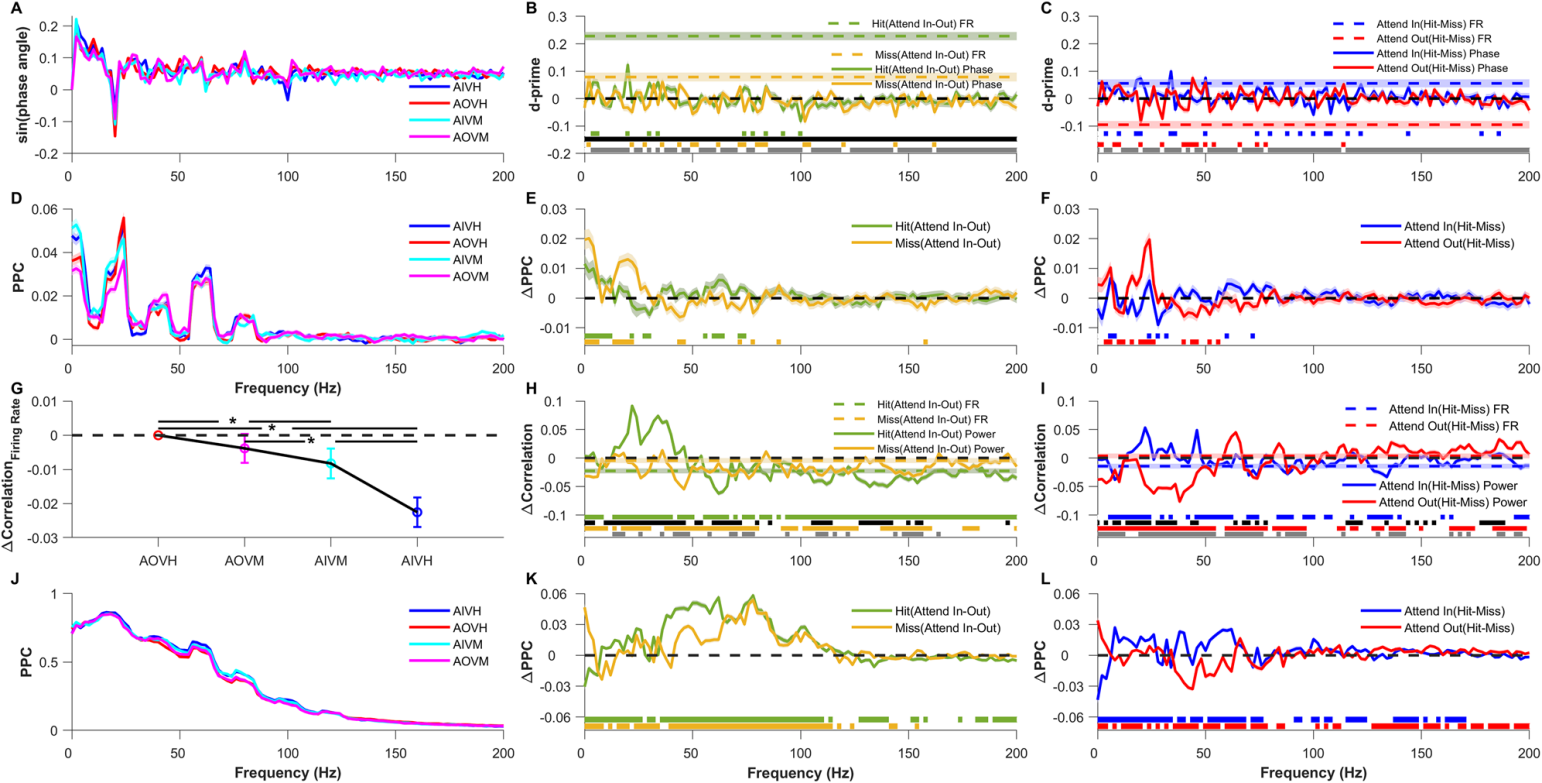
Comparison of LFP phase and pairwise phase consistency of individual electrodes and trial-wise firing rate correlation, LFP power correlation, and pairwise phase consistency (PPC) of electrode pairs across validly cued attention and behavioral conditions. ***A***, Mean sine of the LFP phase angle for Attend-In Valid Hit (AIVH; blue), Attend-Out Valid Hit (AOVH; red), Attend-In Valid Miss (AIVM; cyan), and Attend-Out Valid Miss (AOVM; magenta) conditions. ***B***, Mean *d*′ of firing rate (dashed color lines) and sine of the LFP phase angle (solid lines) between attend-in and attend-out conditions for hit (green) and miss (yellow) conditions. The horizontal dashed black line marks the zero of the *y*-axis. The horizontal green and yellow patches at the bottom indicate the frequencies at which the *d*′ of LFP phase angle for hit and miss conditions, respectively, are significantly greater than zero (Wilcoxon signed-rank test, Benjamini–Yekutieli FDR controlled *p* < 0.05 under unknown dependency). The horizontal black and gray patches at the bottom indicate the frequencies at which the *d*′ of the LFP phase angle is significantly different from the *d*′ of firing rate for hit and miss condition, respectively (Wilcoxon rank-sum test, Benjamini–Yekutieli FDR controlled *p* < 0.05 under unknown dependency). Negative *d*′ values were multiplied by −1 before performing the significance test. ***C***, Mean *d*′ of firing rate (dashed color lines) and sine of the LFP phase angle (solid lines) between hit and miss conditions for attend-in (blue) and attend-out (red) conditions. The horizontal dashed black line marks the zero of the *y*-axis. The horizontal blue and red patches at the bottom indicate the frequencies at which the *d*′ of LFP phase angle for attend-in and attend-out condition, respectively, are significantly greater than zero (Wilcoxon signed-rank test, Benjamini–Yekutieli FDR controlled *p* < 0.05 under unknown dependency). The horizontal gray patches at the bottom indicate the frequencies at which the *d*′ of the LFP phase angle is significantly different from the *d*′ of firing rate for attend-out condition. (Wilcoxon rank-sum test, Benjamini–Yekutieli FDR controlled *p* < 0.05 under unknown dependency). Note that the difference between *d*′ for the LFP phase angle and firing rate for attend-in condition was not significant at any frequency (Wilcoxon rank-sum test, Benjamini–Yekutieli procedure for FDR control under unknown dependency); hence, no patch is shown for that comparison. Negative *d*′ values were multiplied by −1 before performing the significance test. ***D***, Mean single-electrode pairwise phase consistency (PPC; see Materials and Methods for details) for the four conditions as in ***A***. ***E***, Mean change in single-electrode PPC between attend-in and attend-out conditions for hit (green) and miss condition (yellow). The horizontal dashed black line marks the zero of the *y*-axis. The horizontal green and yellow patches at the bottom indicate the frequencies at which the change in PPC for hit and miss condition, respectively, is significantly greater than zero (Wilcoxon signed-rank test, Benjamini–Yekutieli FDR controlled *p* < 0.05 under unknown dependency). ***F***, Mean change in single-electrode PPC between hit and miss conditions for attend-in (blue) and attend-out condition (red). The horizontal dashed black line marks the zero of the *y*-axis. The horizontal blue and red patches at the bottom indicate the frequencies at which the change in PPC for attend-in and attend-out conditions, respectively, are significantly greater than zero (Wilcoxon signed-rank test, Benjamini–Yekutieli FDR controlled *p* < 0.05 under unknown dependency). In ***A***–***F***, mean and SEM are computed like in Figure 2*A,G*. ***G***, Mean change in trial-wise firing rate correlations for the four conditions relative to the Attend-Out Valid Hit (AOVH) condition. The dashed black line indicates the zero of the *y*-axis. The asterisks indicate the conditions between which the differences in trial-wise correlation are significant (Wilcoxon rank-sum test; Bonferroni corrected *p* < 0.05) between the conditions. ***H***, Mean change in trial-wise LFP correlations (solid lines) and firing rate correlation (dashed color lines) between attend-in condition and attend-out condition for hit (green) and miss trials (yellow). The dashed black line indicates the zero of the *y*-axis. ***I***, Mean change in trial-wise LFP correlation (solid lines) and firing rate correlation (dashed color lines) between hit condition and miss condition for validly cued attend-in (blue) and attend-out (red) conditions. The dashed black line indicates the zero of the *y*-axis. ***J***, Mean pairwise phase consistency (PPC) across trials for the four conditions as in (***A***). ***K***, ***L***, Same as ***E*** and ***F*** but for pairwise phase consistency (PPC) across trials. In ***G***–***L***, the mean was taken across 50 bootstrap samples of mean across 5,754 pairs, and the shaded lines and error bar indicate the bootstrap mean of SEM across 5,754 electrode pairs. In ***H***, ***I***, ***K***, and ***L***, the significance lines shown at the bottom are calculated using the same approach as ***B***, ***C***, ***E***, and ***F***. A similar comparison of single-electrode phase measures, trial-wise correlations, and PPC for neutrally cued condition is shown in Extended Data Figure 3-1.

We previously showed that the single-electrode LFP power in the gamma (42–78 Hz) and high-gamma (122–198 Hz) bands performed better than firing rate in decoding attention (Prakash et al., 2021). However, in our present analysis, the power in the gamma and high-gamma ranges did not perform better than firing rate in discriminating the attention condition (Fig. 2*F*, green trace). This discrepancy is due to the variability in the spectral estimator at individual frequency bins (Jarvis and Mitra, 2001), which decreases *d*′ values as observed here. However, spectral variability is uncorrelated across frequencies and therefore can be reduced by summing or averaging the power over the frequencies of interest, which can improve the decoding performance (Prakash et al., 2021). We illustrate this fact by performing similar averaging by smoothening over a frequency window using the multitaper method. The extent of frequency smoothening is controlled by the time-bandwidth product (TW) which determines the number of tapers (*K*) to use (*K* = 2TW − 1) for maximal spectral concentration. Increasing TW increases the frequency window over which the power is smoothed. As expected, as we increased TW, the attentional *d*′ in the gamma and high-gamma bands increased and exceeded the *d*′ of firing rate (Fig. 2*H*). However, smoothing improves performance only when nearby frequencies are modulated in a similar fashion by attention/behavior. For example, note that the enhanced discriminability at SSVEP was eliminated by this smoothing because the nearby frequencies around the SSVEP frequency did not show this discriminability. Thus, frequency smoothing improves the discriminability only when the neighboring frequencies also perform comparably well. The TW parameter is usually chosen depending on the length of the signal and frequency band of interest. For relatively wider frequency bands like gamma and high-gamma choosing TW of five (which smoothens over a frequency bin of ±10 Hz for a 0.5 s signal) or more is appropriate whereas for a narrower band like alpha (8–12 Hz) and SSVEP, the TW should be kept to low values. For a case where different frequency bands of varying bandwidths are used, TW should be chosen such that the wider and narrower frequency activity is captured sufficiently well. We have used TW = 3 (i.e., five tapers) when analyzing the effect of attention/behavior on the power spectrum, as shown in Figures 2, 3. In the final comparison over different frequency bands shown in Figure 6, power is averaged over different frequency ranges prior to the computation of *d*′, which is essentially equivalent to using different numbers of tapers for different frequency ranges depending on the bandwidth of the signal of interest.

The neutral cueing condition showed similar results (compare Fig. 2*A–H* and Extended Data Fig. 2-1*I–P*). The results are shown after matching the target-onset times because we found similar artifacts as the valid condition when target-onset times were not matched (data not shown). As in the valid condition, firing rates in neutral condition were higher when the target appeared inside the RF than when it appeared outside (Fig. 2*A*, Extended Data Fig. 2-1*I* inset), and the ERPs were comparable across conditions (Extended Data Fig. 2-1*J*). However, there were some important differences as well. Firing rates were different for hits versus misses when the target was inside the RF (Extended Data Fig. 2-1*I*, blue and cyan dots that correspond to Target-In Neutral Hit vs Target-In Neutral Miss conditions, respectively), but not when the target was outside the RF (Target-Out Neutral Hit vs Target-Out Neutral Miss). This trend was also reflected in the LFP power, with large discriminability in behavior in the gamma range (which was further comparable to the firing rate discriminability) when the target was in the RF (Extended Data Fig. 2-1*O*, blue trace) but not when outside (Extended Data Fig. 2-1*O*, red trace). The discriminability between target-in and target-out conditions for hit trials was highest in the gamma range and was comparable to the firing rate (Extended Data Fig. 2-1*N*, green trace), and the traces were essentially inverted for the miss condition for both firing rate and LFP power (Extended Data Fig. 2-1*N*, yellow traces). Unlike the valid condition, frequency smoothening did not improve discriminability beyond firing rates (Extended Data Fig. 2-1*P*). Overall, the behavioral discriminability pattern of the neutral cue was similar to the attentional discriminability of the valid condition (in other words, the effect observed in Fig. 2*F* was similar to that in Extended Data Fig. 2-1*O*, while Fig. 2*G* was similar to Extended Data Fig. 2-1*N*).

### Discrimination of attentional state and behavioral outcome using LFP phase

EEG studies have shown that phases at frequency bands such as alpha (8–12 Hz) are modulated by attention and are informative about behavioral performance (Thut et al., 2006; Busch et al., 2009). We therefore examined the ability of the LFP phase to discriminate the attentional and behavioral states. If the LFP phases at which monkeys detect the target are consistently different from the phases at which they miss, the difference should be reflected in the ERPs, which we did not observe (Fig. 2*B*; although note that the presence of strong SSVEP may have affected the endogenous alpha rhythm). Because phase is a circular variable, we used the sine component of the phase vector to make it linear (like firing rate and LFP power) in order to calculate *d*′. This transformation should not affect our results since we are interested in the differences between conditions rather than their absolute values. For valid trials, the phase values were only consistent at the SSVEP frequencies (Fig. 3*A*), but we did not find a consistent difference across conditions at any frequency, resulting in low attentional and behavioral discriminability compared with the firing rate (Fig. 3*B,C*). Similar results were obtained when the cosine component was used instead of the sine component (data not shown).

10.1523/ENEURO.0327-24.2024.f3-1 Figure 3-1 **Comparison of LFP phase and pairwise phase consistency of individual electrodes and trial-wise firing rate correlation, LFP power correlation and pairwise phase consistency (PPC) of electrode pairs across neutrally cued attention and behavioral conditions.** Same as figure 3 but for the neutrally cued conditions. In (A)–(F) mean and s.e.m are computed like in Figure 2-1I – 1O. In (G)-(L) mean was taken across 50 bootstrap samples of mean across 5985 pairs and shaded lines and error bar indicate the bootstrap mean of s.e.m across 5985 electrode pairs. Horizontal color patches at the bottom of each panel indicate the significance level like in Figure 3. Download Figure 3-1, TIF file.

Previous studies have shown that pairwise phase consistency (PPC) across pairs of electrodes, which reflects the consistency of the phase difference between electrodes across trials, varies with attentional condition (Rohenkohl et al., 2018; Prakash et al., 2021). We next tested whether the absolute phase of a single electrode also showed any consistency across trials. For this analysis, we computed the PPC, which is an unbiased estimator of the square of the coherence (Vinck et al., 2010), but replaced the phase difference between a pair of electrodes with the absolute phase of one electrode for each trial. This single-electrode PPC is expected to be high if the absolute phase is consistent across trials. However, PPC was high only at the SSVEP frequency (the peak is seen at 24 Hz instead of 20 due to the smoothening effect of multitaper) and its harmonics (Fig. 3*D*). No consistent trends were observed when we compared across conditions (Fig. 3*E,F*). Note that since PPC is computed across trials, it is not a single-trial measure, and therefore we cannot obtain *d*′ values like above. In summary, the absolute phase of the LFP was much less informative about the attentional location or behavioral state than firing rates, and no difference in the consistency of the phase across trials was observed across conditions. Similar results were obtained for the neutral cue (Extended Data Fig. 3-1*A–F*).

### Effect of attention and behavioral outcome on pairwise measures

Conventional measures based on pairs of electrodes, such as correlation and phase consistency, are computed across trials. Before exploring single-trial estimates of such measures, which are required to estimate discriminability, we evaluated the effects of attention and behavior in the traditional way by computing these measures across trials. Consistent with previous studies (Cohen and Maunsell, 2009; Mitchell et al., 2009; Mayo and Maunsell, 2016), attention reduced correlations in firing rate across pairs of electrodes (Attend-Out Valid Hit vs Attend-In Valid Hit; Fig. 3*G*). The correlation for missed conditions was intermediate to the hit condition (Attend-Out Valid Miss and Attend-In Valid Miss; Fig. 3*G*), consistent with the changes in firing rate as shown in Figure 2*A* inset. Correlations between LFP power recorded from two electrodes increased with attention at SSVEP frequency (20 Hz) and its harmonic but reduced for frequencies higher than 50 Hz for valid hit condition (Fig. 3*H*, green trace). Such modulations were not observed when the animals missed the trials (Fig. 3*H*, yellow trace). Differences between behavioral outcomes (hit vs miss; Fig. 3*I*) were small in the high-gamma range, mirroring the small changes observed in firing rate correlations as well. Interestingly, lower frequencies including the SSVEP frequency showed larger differences for the Attend-out condition (Fig. 3*I*, red trace), which is similar to the strong effect of behavioral differences on SSVEP power observed earlier (Fig. 2*G*). Overall, these results are consistent with previous studies that showed a reduction in spike count correlation (Cohen and Maunsell, 2009; Mitchell et al., 2009) and LFP power correlation except at SSVEP (Prakash et al., 2021).

Next, we analyzed the PPC between the LFP signals from pairs of electrodes (Fig. 3*J–L*). PPC increased for valid condition when attention was cued into the RF over a broad range of frequencies between 36 and 106 Hz (Fig. 3*K*, green trace), although we did not observe much difference beyond 120 Hz even though LFP power was elevated in that range (compare with Fig. 2*D*, green trace). The reason for this difference between high-frequency power and PPC could be the poor sensitivity of phase synchronization measures at higher frequencies (Ray, 2022). Interestingly, PPC was more sensitive to attentional differences (Fig. 3*K*) than behavioral differences (Fig. 3*L*).

For neutral condition (Extended Data Fig. 3-1*G–L*), the effect of target location (Extended Data Fig. 3-1*H,K*) was somewhat similar to the behavioral effect of the valid condition (Fig. 3*I,L*), and the behavioral effect (Extended Data Fig. 3-1*I,L*) was similar to the attentional effect of the valid condition (Fig. 3*H,K*), as observed previously with LFP power (Extended Data Fig. 2-1*N* is similar to Fig. 2*G* with the two traces flipped horizontally, while Extended Data Fig. 2-1*O* is similar to Fig. 2*F* with one trace showing salient effect and the other one close to zero).

### Discrimination of attentional state and behavioral outcome using pairwise measures

**Figure 4.**
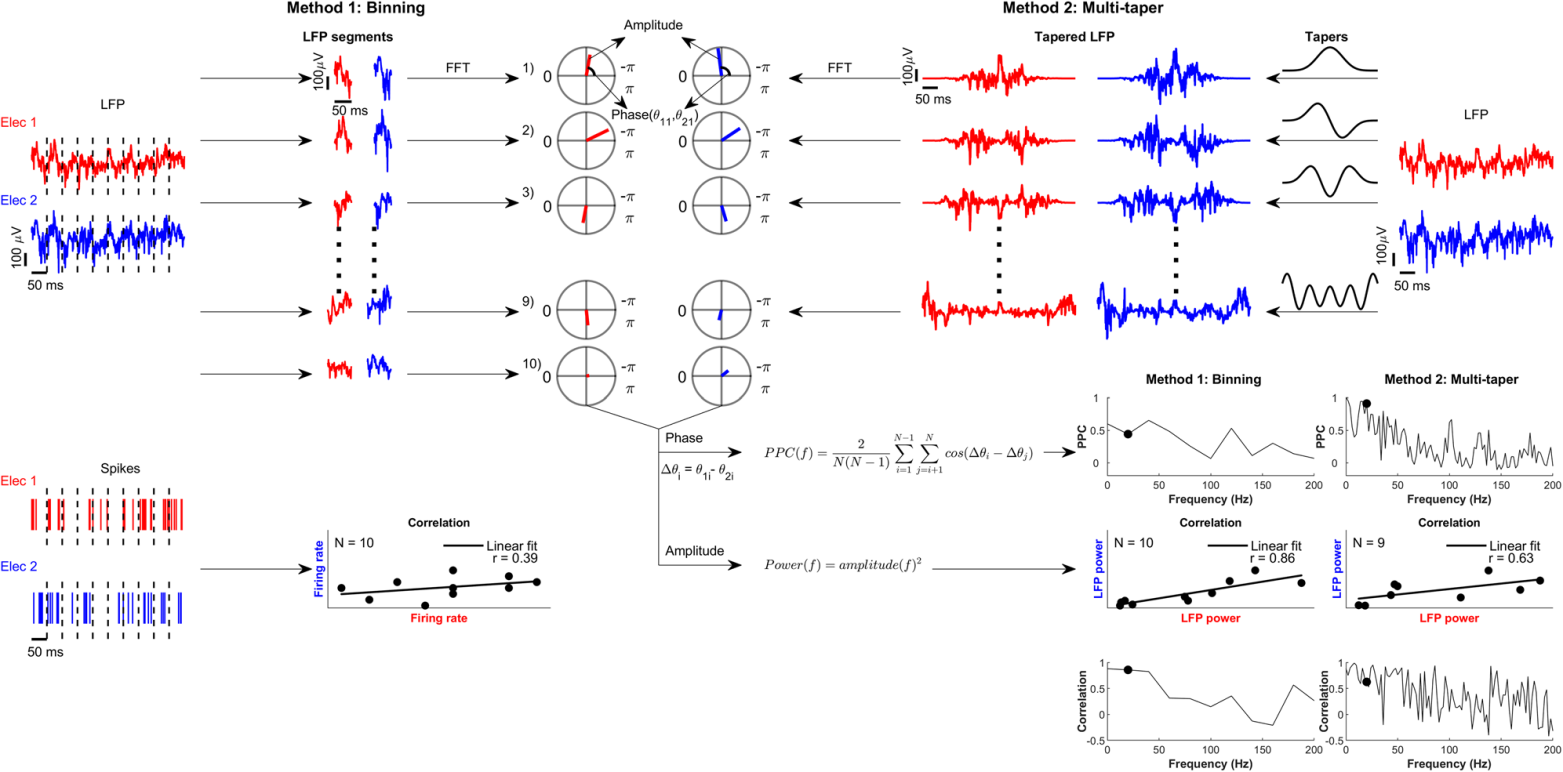
Illustration of methods used for estimating correlation and pairwise phase consistency within a trial. Binning method (left panel), A single-trial LFP signal of 500 ms duration from two electrodes is first divided into 10 nonoverlapping bins of 50 ms each. Then, the power and phase of each 50 ms LFP segment of the two electrodes were estimated by Fourier transform. Subsequently, we estimated the power correlation and pairwise phase consistency across the ten bins between the pair of electrodes at each frequency between 0 and 200 Hz with 20 Hz resolution. Similarly, the spikes from the two electrodes were also binned and firing rate correlations between the two electrodes were computed across bins (bottom left panel). These correlations were subsequently shuffle corrected (see Materials and Methods). Multitaper method (right panel), We used an alternate method to obtain multiple estimates of power and phase using the multitaper method. LFP signal of 500 ms duration of two electrodes was first multiplied by nine orthogonal Slepian tapers of the same length, and then power and phase of these nine tapered LFP signals were obtained by Fourier transform. Power correlation and PPC were calculated between electrode pairs across tapers at frequencies between 0 and 200 Hz with 2 Hz resolution. The black dot in the PPC vs frequency and correlation versus frequency plots indicates the frequency whose amplitude and phase are shown in the polar plot in the top panel and whose power scatter plot is shown.

Modulation of pairwise measures by attentional state and behavioral outcome motivated us to develop methods to obtain the same measures in single trials, which could then be used to obtain discriminability values (*d*′) like the power and phase measures we used earlier. We devised two independent methods to estimate correlation and phase consistency within a single trial. In the first method, we divided the spiking and LFP signal of a single trial into ten nonoverlapping bins of 50 ms each and computed the firing rate, LFP power, and phase in each bin. We then calculated the correlation and LFP–LFP phase consistency between electrode pairs across bins (Fig. 4, left panel). In the second approach, we used the multitaper method where a single-trial LFP signal was multiplied by nine orthogonal Slepian tapers to obtain nine independent estimates of LFP power and phase (Fig. 4, right panel) and computed their correlation and phase consistency (see Materials and Methods for more details). The first method is more direct because power and LFP values are obtained from nonoverlapping segments of data, although the frequency resolution (reciprocal of the analysis duration in seconds) becomes 20 Hz. The second method uses the same length of data (500 ms) to get several estimates of power and phase (which are independent because the tapers are orthogonal) and therefore has a frequency resolution of 2 Hz. We also used a method based on the Hilbert transform method to estimate instantaneous power and phase at each time point within a trial and then computed correlation and phase consistency across time (Spyropoulos et al., 2024).

**Figure 5.**
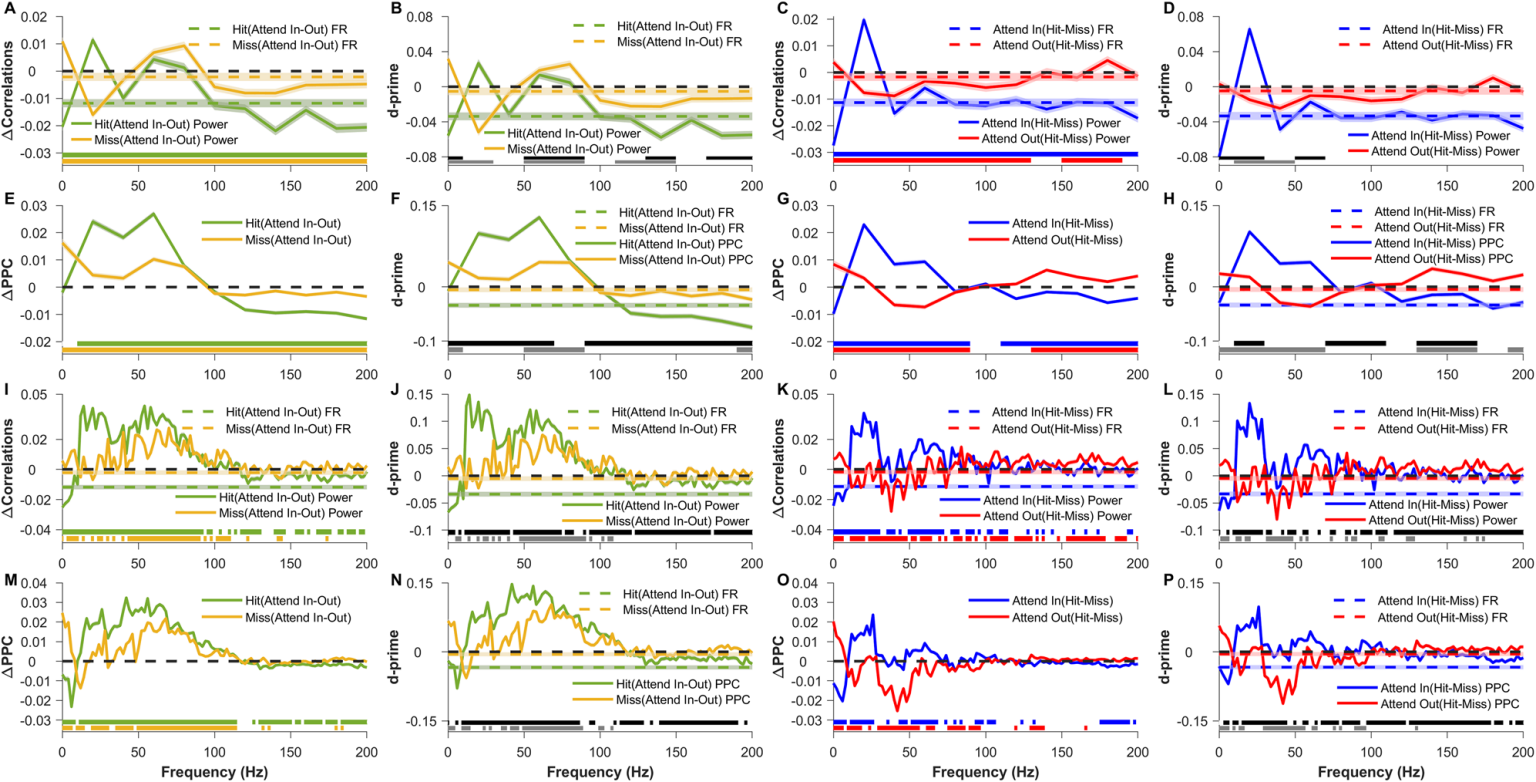
Comparison of single-trial bin-wise firing rate correlation, bin-wise and taper-wise LFP power correlation, and pairwise phase consistency (PPC) across validly cued attention and behavioral conditions. ***A***, Mean change in single-trial bin-wise LFP power correlation (solid lines) and firing rate correlation (dashed color lines) between validly cued attend-in and attend-out conditions for hit (green) and miss (yellow) conditions. Single-trial bin-wise correlation was measured by dividing the analysis period of 500 ms into 10 nonoverlapping bins and measuring the firing rate or power correlation across bins. Since power was estimated for signals of 50 ms duration, the frequency resolution is 20 Hz. The dashed black line indicates the zero of the *y*-axis. ***B***, Mean *d*′ of bin-wise LFP power correlation (solid lines) and firing rate correlation (dashed color lines) between validly cued attend-in and attend-out conditions for hit (green) and miss (yellow) conditions. The dashed black line indicates the zero of the *y*-axis. ***C***, Mean change in bin-wise LFP power correlation (solid lines) and firing rate correlation (dashed color lines) between hit and miss conditions for validly cued attend-in (blue) and attend-out (red) conditions. The dashed black line indicates the zero of the *y*-axis. ***D***, Mean *d*′ of bin-wise LFP power correlation (solid lines) and firing rate correlation (dashed color lines) between hit and miss conditions for validly cued attend-in (blue) and attend-out (red) conditions. The dashed black line indicates the zero of the *y*-axis. ***E***–***H***, Same as ***A***–***D*** but for single-trial bin-wise PPC. Single-trial bin-wise PPC was computed across the same 10 nonoverlapping bins used to compute power correlations. In ***A***–***H***, the colored patches at the bottom indicate significance like that in Figure 3, but here the FDR was controlled using the Benjamini–Hochberg procedure. ***I***–***P***, Same as ***A***–***H*** but for single-trial taper-wise power correlation and PPC. Here power correlation and PPC are computed by using the multitaper method. Specifically, we use TW = 5 to get 9 (2TW-1) estimates of power and phase values per trial and compute the power correlation or PPC between electrode pairs across these nine values. Unlike ***A***–***H***, FDR was controlled by using the Benjamini–Yekutieli procedure under unknown dependency. In ***A***–***P***, the mean was taken across 50 bootstrap samples of mean across 5,754 pairs and shaded lines indicate the bootstrap mean of SEM across 5,754 electrode pairs. A similar comparison of single-trial measures for neutrally cued condition computed using the binning method and multitaper method are shown in Extended Data Figure 5-1 and those computed using the Hilbert transform method for valid and neutral conditions are shown in Extended Data Figure 5-2.

Firing rate correlations were reduced with attention for hit trials even when computed over a single trial (Fig. 5*A*, dashed green line), but not reduced in miss trials (Fig. 5*A*, dashed yellow line), consistent with the across-trial correlation measures (Fig. 3*H*). Because these were single-trial measures, we could now calculate and compare the discriminability by calculating *d*′ values in the same way as before (Fig. 5*B*). LFP correlations in the high-gamma range could distinguish the attention conditions slightly better than firing rate correlation when the targets were correctly detected (Fig. 5*B*, green trace), although these *d*′ values were considerably lower than the ones obtained using firing rate/LFP power (compare with similar traces in Fig. 2*F*). Similar trends were obtained for behavioral comparison: (1) magnitude of discriminability (Fig. 5*D*) was higher in the high-gamma range where it was comparable to firing rate correlations for the attend-in condition (Fig. 5*D*, blue traces), and (2) for the attend-out condition, the low-gamma frequencies were more discriminable (Fig. 5*D*, red trace) than the firing rate for that condition. In summary, we found that the discriminability of high-gamma and firing rate correlations were comparable. Interestingly, the magnitude of *d*′ values of bin-wise PPC was considerably higher (Fig. 5*F,H*) compared with that of correlations in firing rate/power, suggesting that single-trial PPC, especially in the gamma range, was a useful measure to discriminate attention or the behavioral outcome.

10.1523/ENEURO.0327-24.2024.f5-1 Figure 5-1 **Comparison of single trial bin-wise firing rate correlation, bin-wise and taper-wise LFP power correlation and pairwise phase consistency (PPC) across neutrally cued attention and behavioral conditions.** Same as figure 5 but for neutrally cued conditions. Mean and s.e.m were computed like in Figure 3-1G – L. Horizontal color patches at the bottom of each panel indicate the significance level like in Figure 5. Download Figure 5-1, TIF file.

10.1523/ENEURO.0327-24.2024.f5-2 Figure 5-2 **Comparison of single trial estimates of LFP power correlation and pairwise phase consistency (PPC) computed using Hilbert transform method with bin-wise firing rate correlation across valid and neutrally cued attention and behavioral conditions.** (A)-(H) Same as figure 5I - P. Mean was taken across 50 bootstrap samples of mean across 5754 pairs and shaded lines indicate the bootstrap mean of s.e.m across 5754 electrode pairs. Horizontal color patches at the bottom of each panel indicate the significance level like in Figure 5. (I)-(P) Same as figure 5-1I-P. Mean was taken across 50 bootstrap samples of mean across 5985 pairs and shaded lines indicate the bootstrap mean of s.e.m across 5985 electrode pairs. Horizontal color patches at the bottom of each panel indicate the significance level like in (A)-(H). Download Figure 5-2, TIF file.

Although the binning method showed some discriminability, it was inherently limited in its frequency resolution. To overcome this limitation, we used the multitaper method and computed correlation and PPC across tapers (Fig. 4, Method 2). The binning and multitaper methods produced similar results, validating our approaches. For correlations (Fig. 5*I–L*), while the trends were generally similar to the bin-wise approach (compare with Fig. 5*A–D*), we found two important differences. First, while the high-gamma band beyond 100 Hz essentially reflected the trends observed in firing rates for the bin-wise method (Fig. 5*A*), the multitaper method produced negligible correlation differences above 100 Hz (Fig. 5*I*). On the other hand, the difference in LFP correlations between the conditions improved remarkably in the gamma band and below (<60 Hz) for taper-wise method, and hence the discriminability index increased threefold compared with the binning method (compare Fig. 5*B,D* with Fig. 5*J,L*; note the difference in scales). In contrast, the PPC measure (Fig. 5*M–P*) remained comparable across both methods (Fig. 5*E–H*). Among the electrode pair measures, PPC in the low-frequency to mid-frequency range was the most useful measure to discriminate attention and behavior. The neutral conditions (Extended Data Fig. 5-1), reflected the same results as observed for valid trials. Single-trial estimates of power correlation and phase consistency calculated using the Hilbert transform method (Extended Data Fig. 5-2) yielded largely comparable results.

**Figure 6.**
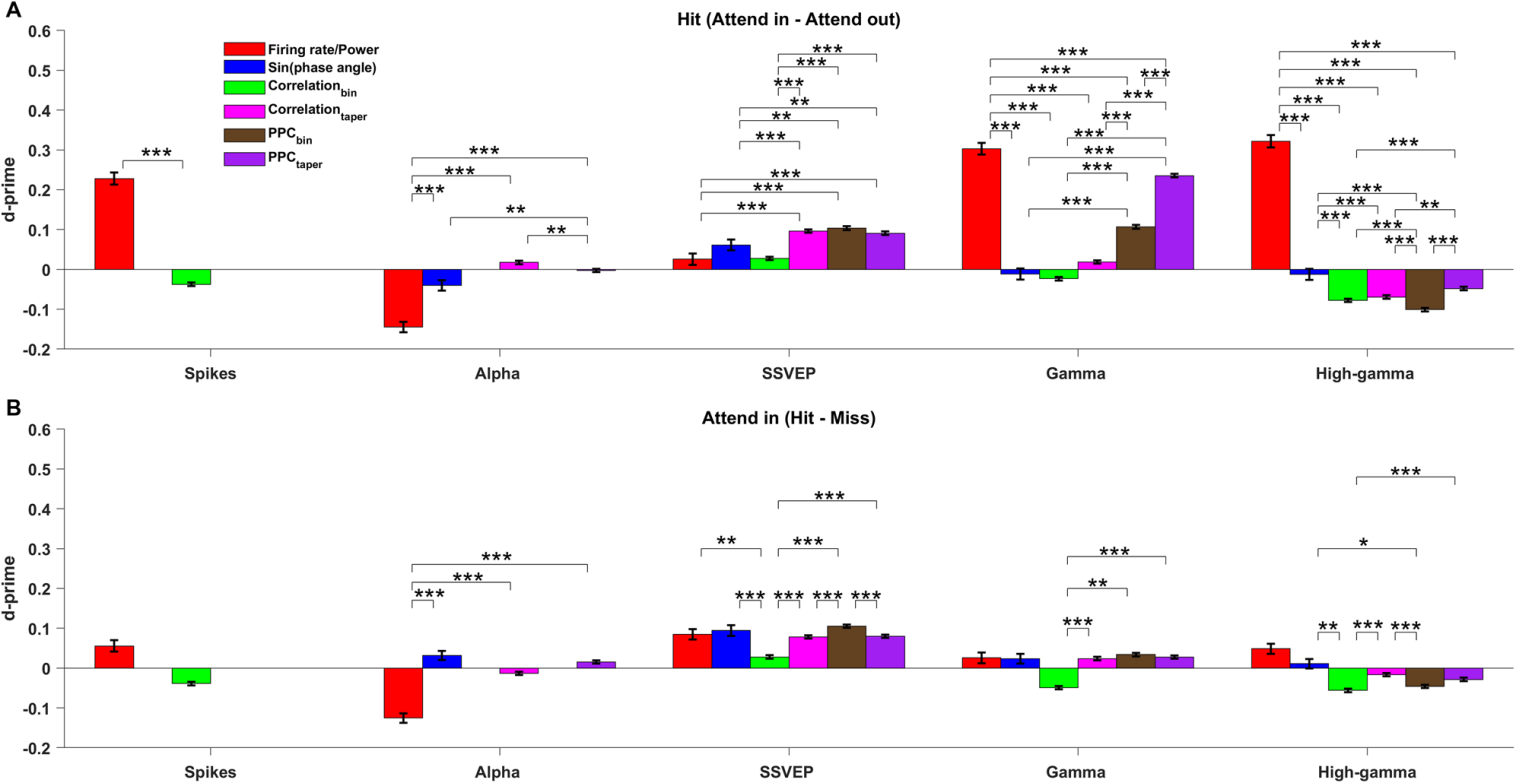
Summary plot comparing across the *d*′ values of all the measures for spikes, LFP power, and phase in different frequency bands for validly cued attention and behavioral comparison. ***A***, *d*′ of firing rate/LFP power (red), sine of the phase angle (blue), firing rate/LFP power correlation across the bins (green), LFP power correlation across the tapers (magenta), pairwise phase consistency (PPC) across bins (brown), and PPC across tapers (purple) for spikes, alpha (8–12 Hz), SSVEP (18–22 Hz), gamma (40–80 Hz), and high-gamma (120–200 Hz) power between attend-in and attend-out conditions for hit trials. Note that for spikes, only *d*′ values of firing rate and bin-wise correlation exist because the rest of the measures cannot be computed for the spike data whereas for alpha-band, *d*′ of bin-wise measures (Correlation_Bin_ and PPC_Bin_) are absent because the frequency resolution in the binning method was 20 Hz. “***” indicates *p*-value < 0.001, “**” indicates 0.001 < *p*-values < 0.01, “*” indicates the 0.01 < *p*-values < 0.05, and no asterisks are shown for *p*-values > 0.05. Negative *d*′ values were converted to positive values by multiplying with −1 before performing the significance test since we were comparing only the magnitude. All the *p*-values are Bonferroni corrected for multiple comparisons. ***B***, Same as ***A*** but for the *d*′ between the hit and miss conditions of the attend-in condition. A summary plot comparing the *d*′ values of all the measures for neutrally cued condition is shown in Extended Data Figure 6-1.

Finally, because our analyses in Figure 2*H* demonstrated that discriminability differs as a function of smoothening over frequency ranges, we computed all the LFP measures within the frequency bands of alpha (8–12 Hz), SSVEP (18–22 Hz), gamma (40–80 Hz), and high-gamma (120–200 Hz) and then computed their *d*′ values. Note that the *d*′ values obtained this way are not similar to the simple average of *d*′ values of individual frequencies computed earlier (Figs. 2, 5). There are two reasons for this. First, as discussed above, averaging the LFP power across frequencies reduces the variability due to the spectral estimator which improves *d*′, as shown in Figure 2*H* where increasing the number of tapers increases *d*′. Second, the total power in a band is dominated by lower frequencies within the band, since power falls with increasing frequency, and hence the *d*′ values are biased toward that of lower frequencies rather than reflecting average *d*′. We summarized our results in Figure 6. The same results for the neutral condition are shown in Extended Data Figure 6-1. Our key findings are summarized as follows: (1) LFP power in the high-gamma and gamma bands were best suited for discriminating “attend-in” versus “attend-out” attentional state (*d*′ of 0.32 ± 0.01 and 0.3 ± 0.01; Fig. 6*A*). Firing rate was the next most informative measure (*d*′ of 0.23 ± 0.01; Fig. 6*A*), but discriminability using gamma was significantly greater than that of firing rate (*p* = 4.6 × 10^−5^; Wilcoxon rank-sum test). Alpha power also had strong discriminability (*d*′ of −0.14 ± 0.01), although the magnitude was significantly less than the firing rate (*p* = 1.1 × 10^−6^; Wilcoxon rank-sum test). (2) While gamma, high-gamma, and spiking activity could all be reliably used to discriminate attentional state, alpha power, SSVEP power, and SSVEP phase were the most informative measures for discriminating behavioral outcome (Fig. 6*B*). In general, firing rates and high-frequency LFP (gamma and high-gamma) were not very useful to discriminate behavior. (3) Among the phase-based measures, single-trial PPC in the gamma band was the most informative of the attentional state, especially when calculated using the multitaper method (*d*′ of 0.23 ± 0.004; Fig. 6*A*, purple bar), on par with the firing rate (*p* = 0.95; Wilcoxon rank-sum test). (4) The performance of the neural measures in the discriminating behavioral outcome for neutral condition (Extended Data Fig. 6-1*B*) was similar to the discrimination of attentional conditions for valid condition (Fig. 6*A*).

10.1523/ENEURO.0327-24.2024.f6-1 Figure 6-1 **Summary plot comparing across the d-primes of all the measures for spikes, LFP power and phase in different frequency bands for neutrally cued attention and behavioral comparison** Same as figure 6 but for neutrally cued condition. Asterisks indicate the significance level like in Figure 6. Download Figure 6-1, TIF file.

## Discussion

We studied the effect of attentional (cue inside vs outside RF) location and behavioral outcome (hit vs miss) on distinct frequency bands of the LFP. Our analyses illustrated potential issues caused by visual transients that can differentially affect measures of behavioral outcome, which could be corrected by matching the target-onset distributions for different conditions. While high-frequency LFP power in the gamma (42–78 Hz) and high-gamma (122–198 Hz) band decoded the attentional state best, low-frequency power in the alpha (8–12 Hz) and SSVEP (18–22 Hz) range decoded the behavior well. We also used single-trial estimates of correlation and pairwise phase consistency (PPC) which showed similar attention and behavioral modulations as their trial-wise counterparts. Although these metrics did not produce the best decoding performance in our dataset, they will likely be useful for future investigations of the dynamics of attention. Phase-based measures performed poorly compared to power-based measures. Nonetheless, among the phase-based measures, taper-wise PPC in the gamma band was the best, performing on par with the firing rate in decoding attention.

### Comparison with previous studies

LFP high-gamma power has been shown to reflect the average firing rate of the neuronal population around the microelectrode tip (Ray et al., 2008; Ray and Maunsell, 2011). Unlike LFP, the increase in high-gamma power in electrocorticogram (ECoG)—a signal that integrates over a larger population than LFP—cannot be accounted solely by increases in firing rate and has been hypothesized to reflect more synchronous or correlated activity in the neural population (Ray et al., 2008). Interestingly, we found that attentional discriminability of LFP high-gamma power was higher than that of firing rate which suggests that, like ECoG, high-gamma power of LFP could also partially reflect firing rate correlations and synchrony (in addition to the average firing rate) which also get modulated with attention (Cohen and Maunsell, 2009). In addition, improved performance of higher LFP frequencies could simply be due to optimal averaging of responses in the LFP signal over the spatial scale over which attention operates (Prakash et al., 2022).

Our results are consistent with the findings of human EEG studies (Thut et al., 2006; Hanslmayr et al., 2007; Busch et al., 2009) which showed that alpha power was lower when subjects detected a visual target than when they missed it, although we note that the endogenous alpha rhythm in our study could be compromised because of the strong 10 Hz counterphasing stimulus. However, care should be taken to match the distribution of target-onset times for hit and miss trials while analyzing the data in the pretarget period to avoid the effect of stimulus onset-related transients that can artificially inflate the behavioral effects on low-frequency power, especially in a change detection task which is often used in attention studies.

We found the performance of phase-based measures underwhelming given many studies have shown the importance of phase in attention and behavioral performance (Busch and VanRullen, 2010; Fiebelkorn et al., 2018; Rohenkohl et al., 2018). For example, attention improved the consistency of gamma-band phase relations between two neuronal groups, which is thought to be crucial for the effective relay of information (Fries et al., 2001). Attention is also suggested to operate in a rhythmic manner which is mediated by the phase of alpha and theta band (Busch and VanRullen, 2010; Landau and Fries, 2012; Fiebelkorn et al., 2018), although a recent study has questioned these results (Brookshire, 2022). Previous LFP studies that used reaction time as the behavioral readout have shown that absolute phase (Ni et al., 2016) and phase coherency (Womelsdorf et al., 2006) in the gamma band are predictive of behavior. Despite these mechanistic implications of phase on attention, we did not find any effect of absolute phase. Even with pairwise measures, only gamma-band PPC managed to perform well in discriminating attentional state, although not as well as power-based measures. Interestingly, the attentional discriminability of gamma-band PPC was comparable to that of firing rate, contrary to a recent finding where the V4 firing rate decoded attention better than interareal gamma-band PPC between V1 and V4 (Spyropoulos et al., 2024). Furthermore, several EEG studies (Busch and VanRullen, 2010; Harris et al., 2018), showed that the detection performance at the cued location systematically depended on the phase of the alpha-band, and their performance in discriminating the behavioral outcome was marginal. The performance of SSVEP phase measures, on the other hand, was comparable to that of its power. Our results of LFP power-based measure performing better than phase-based measures are consistent with a similar finding in epidural ECoG recordings in macaque visual areas V1 and V4 (Rotermund et al., 2013).

In our study, the absolute phase of LFP did not perform well in discriminating attention or behavioral outcome, possibly because the phase values were estimated over a coarser timescale of 500 ms, during which phase values at higher frequencies were repeated multiple times. High-frequency oscillations often occur in short bursts (Feingold et al., 2015; Lundqvist et al., 2016); for example, gamma oscillations occur in bursts with a duration of 100–300 ms (Burns et al., 2011; Xing et al., 2012; Chandran et al., 2018), such that a single phase value estimated over 500 ms may not capture the changes occurring over shorter timescales. We minimized this limitation by calculating phase over a shorter time period of 200 ms before the target onset, but results did not change appreciably (data not shown). Further, earlier studies (Busch et al., 2009; Ni et al., 2016; Harris et al., 2018) have compared absolute phases for different behavioral measures using different techniques. For example, in previous studies (Busch et al., 2009; Harris et al., 2018), intertrial coherence (ITC)—a measure that quantifies the concentration of phase vectors across trials—was computed separately for trials of hit and miss conditions. These ITC values were then compared against the overall ITC computed across all the trials from both conditions combined to quantify the differences in phase concentration as well as the average phase value between the two conditions. This is essentially a trial-averaged measure, which is different from using the sine or cosine of the phase angle of individual trials as done in this study since our main goal was to compute *d*′ values for all measures for a common comparison, which cannot be computed for previous methods.

### Shared and distinct effects of attention and behavior on LFP

Although subjects are better at detecting the target when it appears at the cued location, they occasionally miss the target. If this is due to the misallocation of attention, the modulation of neural activity by attention (attend-in vs attend-out) and behavioral outcome (hit vs miss) should be similar, which was not the case. One reason could be that in a missed trial, the overall attentional (or arousal) level could be low (rather than focused on the incorrect side), which would further be reflected in the power of low-frequency rhythms such as alpha (Hong et al., 2014; van Kempen et al., 2019; Johnston et al., 2022). Further, a behavioral report involves a decision variable and hence relates to different aspects such as bias or criterion, as compared with changes in sensitivity (Sridharan et al., 2014; Banerjee et al., 2019; Luo and Maunsell, 2019). Some studies have suggested that bias could be reflected in alpha power (Limbach and Corballis, 2016; Iemi et al., 2017; Samaha et al., 2020; but see Podvalny et al., 2021; Zhou et al., 2021), potentially leading to better discriminability of behavioral outcome using alpha power. Saccade preparation is another factor that can modulate the low-frequency oscillations in V4 (Steinmetz and Moore, 2014), that might contribute to better behavioral discriminability. However, a recent study that used the same experimental data found no difference in the microsaccade direction—a potential indicator of saccade preparation (Hafed and Krauzlis, 2012)—between hits and miss trials (Willett and Mayo, 2023).

Our results also relate to the potential roles of fast (gamma) and slow (alpha/beta) oscillations in mediating feedforward and feedback communication across brain areas, as proposed by some studies (van Kerkoerle et al., 2014; Bastos et al., 2015). This notion implies that attentional modulation somehow affects the feedforward pathway while behavioral outcome (target detection) affects feedback, which is unlikely for several reasons. First, attention is shown to enhance gamma-band causal influence (Granger causality) in both feedforward and feedback directions between areas V4 and V1 and within V4 (Ferro et al., 2021). Second, attention signals are shown to originate from higher areas like the frontal eye field (FEF) which sends long-range feedback connections to V4 (Gregoriou et al., 2009). On the other hand, the behavioral outcome effects could be mediated through the feedback pathway because an enhancement of alpha-band activity was observed in the lateral intraparietal area when the perceptual sensitivity was low which can lead to a miss and a boost in the beta band activity in FEF when the perceptual sensitivity was high which can lead to a hit (Fiebelkorn et al., 2018). The interareal coherence usually increases in the frequency bands that have higher power (Schneider et al., 2021), and consequently, the behavioral outcome information can propagate through the low-frequency mediated feedback signal to lower areas like V4. Therefore, our results show that modulation of high- or low-frequency oscillations might not always result from the exclusive engagement of feedforward or feedback pathways, respectively, but instead can arise from both feedforward and feedback, at least for attentional effects.

### Decoding performance in the neutral condition

There were similarities as well as stark differences in discriminability of attention and behavioral outcomes in neutral condition. Like in valid condition, LFP power was better at decoding than phase measures. However, high-frequency oscillations were better in decoding behavioral outcome than attention location, in contrast to valid condition. The difference in the discriminability pattern could be due to the differences in the strategies employed in the two cue conditions. In the neutral condition, subjects likely had to divide their attention or switch between the two locations since the target was equally likely to appear at either location. The similarity between the decoding pattern of behavioral outcome (hit vs miss) in neutral condition and attention (attend-in vs attend-out) in valid condition suggests that the focus of attention was outside the receptive field whenever the subjects missed the target that appeared inside the receptive field, since the target was equally likely to occur on either side, making it similar to the attend-in and attend-out of the valid condition. This explanation holds only if the subjects were switching between the two locations. However, we do not know the exact strategy the subjects used during neutral cue; future experiments that control for the strategies employed by the subject may help resolve the similarities observed between the decoding patterns of different states of the valid and neutral conditions.

Overall, our results suggest that LFP carries attention and behavior-related information in distinct frequency bands. This distinction may implicate the role of a decision-related variable such as bias in influencing behavioral outcome in addition to sensory or perceptual effects of attention. Another striking observation was the similarity between the attentional and the behavioral discriminability of the valid and neutral condition which hints at the underlying strategies employed in the two different cueing conditions. Surprisingly, despite their proposed implications in attention and behavior, phase-based measures were not an optimal choice to discriminate attention location and behavioral outcome.

## Synthesis

Reviewing Editor: Laura Bradfield, University of Technology Sydney Decisions are customarily a result of the Reviewing Editor and the peer reviewers coming together and discussing their recommendations until a consensus is reached. When revisions are invited, a fact-based synthesis statement explaining their decision and outlining what is needed to prepare a revision will be listed below. The following reviewer(s) agreed to reveal their identity: NONE.

Thank you for submitting your manuscript to eNeuro. The reviewers and I have discussed your manuscript and have determined that it needs significant re-writing in order to make it less complicated and more accessible for readers. Although the abstract, introduction and methods are mostly written quite clearly, we found the results - both in the text and figures, difficult to follow. Our specific recommendations are:

1. Please ensure the figures are edited carefully for clarity, and in particular please make sure that they match the summary Figure 6.

2. The results in Figure 3 and Figure 5 do not seem to align with what is written in the text. Please revise.

3. Please reduce jargon and avoid acronyms unless they are well-known in the field (e.g. LFP for local field potential) or are abbreviating overly complex or unusual terms. Using too many acronyms, particularly for words that are commonly known (e.g. PC for phase continuity) can reduce readability. Please consider readers outside of your field when revising.

Outside of these suggestions for revising the text, please also take into account the specific comments of each reviewer, particularly with regards to whether the smoothening over frequency ranges is a valid approach for discriminating states, and please make sure all claims are statistically supported.

The specific reviews are appended:

Reviewer #1

General summary:

The authors investigated how local-field potential (LFP) recorded from V4 in primates encoded attentional conditions and behavioral outcomes separately. By using standard methods, they are able to extract the features of LFP such as phase and power from different frequency bands, and it allows them to calculate the standardized differences for comparisons within or between task conditions. They concluded that the attentional conditions are better decoded by high frequency bands such as high-gamma and gamma band, whereas the behavioral outcomes are better decoded by low frequency bands such as alpha band and the steady-state-visually-evoked-potential (SSVEP).

I do think this paper has made great efforts and contributions to the field, especially for examining the distinct types of LFP signals responded to attentional states and behavioral outcomes separately. This would encourage the researchers using electrophysiology in primates to study the brain functions by extracting more features of LFP signals.

My main concern is about the statistical testing for the comparisons between methods or signal types. The comments are listed below. I would be happy to read a revised paper, but this could also be handled at the editorial level.

Major comments:

1) Line 386-389: The conclusion of the effects of frequency smoothing is clear by Figure 2H, suggesting that frequency smoothing improved the discriminability. However, the notice "enhanced discriminability at SSVEP was eliminated by this smoothing" sounds against the conclusion. I may miss some links between, does it indicate the effects of frequency smoothing work across frequencies except for SSVEP? If the smoothing effects are controlled by time-bandwidth product, how do you know which parameters are appropriate? Could you provide more details?

2) In Line 496-519, the results from most panels in Figure 5 are not easy to compare back to Figure 2 and Figure 3 by vague descriptions. They may show similar patterns or trends, but it is still too abstract to be convinced by eyeballing with no statistical testing, e.g. yellow traces in Figure 5A and 3H. If possible, it would be great to show statistical tests and results before making conclusions. It seems to me that these two paragraphs did not provide more information than Figure 6 if there is no statistical tests to support the descriptions.

Minor comments:

1) Line 65: typos of citations in the sentence.

2) Line 282: it may be a typo in "Figure 2-11A-G", because there is no Figure 2-11.

3) It would be clearer for readers to have distinct labels for main figures and extended figures. For example, using "Fig. 2E and Extended Fig. 2E" is better than "Fig. 2E and Fig. 2-1E."

3) Line 790 and 829: the citations are incomplete.

Reviewer #2

The paper is written in a complicated manner. The relevance of gamma and high gamma in the behavioural tasks is not clear. The authors have consistently referrred a single paper by Prakash et al for throughout the manuscript, how this study is an advancement in this field and would further help to understand the mechanism of attention and behaviour is lacking.

## Author Response

Synthesis of Reviews:

Synthesis Statement for Author (Required):

Thank you for submitting your manuscript to eNeuro. The reviewers and I have discussed your manuscript and have determined that it needs significant re-writing in order to make it less complicated and more accessible for readers. Although the abstract, introduction and methods are mostly written clearly, we found the results - both in the text and figures - difficult to follow. >> We thank the reviewers and reviewing editor for their constructive feedback and recommendations. We have now made significant changes in the manuscript to address the concerns.

Our specific recommendations are:

1. Please ensure the figures are edited carefully for clarity, and in particular please make sure that they match the summary Figure 6. >> Thank you for the recommendation. The summary Figure 6 varies slightly from rest of the figures in terms of d-prime values because for Figure 6, we computed the d-prime for the LFP measures after first averaging the power across a frequency band. This is different from computing the d-prime at individual frequencies (as done in other figures) and then taking the average of the d-primes across the frequency band. There are two reasons for this. First, averaging the LFP power across frequencies reduces the variability due to the spectral estimator which improves d-prime, as shown in Figure 2H where increasing the number of tapers increased d-prime. Second, the total power in a band is dominated by lower frequencies within the band, since power falls with increasing frequency, and hence the d-prime values are biased towards that of lower frequencies rather than reflecting average d-prime. We have made this clear in the last paragraph of the result section.

2. The results in Figure 3 and Figure 5 do not seem to align with what is written in the text. Please revise. >> We thank for the recommendation. We agree that the analogy between Figure 3 and 5 was descriptive and did not add much value beyond what can be seen in Figure 6. We have therefore removed the lines which qualitatively described the similarity between Figure 5 and Figure 3.

3. Please consider readers outside of your field when revising. For instance, please reduce jargon and avoid acronyms unless they are well-known in the field or are abbreviating overly complex or unusual terms. Using too many acronyms reduces readability. >> We thank for rising this important point. We have tried to improve the readability of the paper by removing the abbreviations used for different attention and behavioral conditions. We have also removed few sentences that may appear complicated so that text is concise and clearer to the general readers preserving the structure and main results of the paper.

Outside of these suggestions for revising the text, please also take into account the specific comments of each reviewer. In particular, please determine whether the smoothening over frequency ranges is a valid approach for discriminating states, and please make sure all claims are statistically supported.

The specific reviews are appended:

Reviewer #1 General summary:

The authors investigated how local-field potential (LFP) recorded from V4 in primates encoded attentional conditions and behavioral outcomes separately. By using standard methods, they are able to extract the features of LFP such as phase and power from different frequency bands, and it allows them to calculate the standardized differences for comparisons within or between task conditions. They concluded that the attentional conditions are better decoded by high frequency bands such as high-gamma and gamma band, whereas the behavioral outcomes are better decoded by low frequency bands such as alpha band and the steady-state-visually-evoked-potential (SSVEP).

I do think this paper has made great efforts and contributions to the field, especially for examining the distinct types of LFP signals responded to attentional states and behavioral outcomes separately. This would encourage the researchers using electrophysiology in primates to study the brain functions by extracting more features of LFP signals. >> We thank the reviewer for the encouraging comments.

My main concern is about the statistical testing for the comparisons between methods or signal types. The comments are listed below. I would be happy to read a revised paper, but this could also be handled at the editorial level.

Major comments:

1. Line 386-389: The conclusion of the effects of frequency smoothing is clear by Figure 2H, suggesting that frequency smoothing improved the discriminability. However, the notice "enhanced discriminability at SSVEP was eliminated by this smoothing" sounds against the conclusion. I may miss some links between, does it indicate the effects of frequency smoothing work across frequencies except for SSVEP? If the smoothing effects are controlled by time-bandwidth product, how do you know which parameters are appropriate? Could you provide more details? >> We apologize for the confusion. The main point is that frequency smoothening improves the discriminability only if the neighboring frequencies are similarly modulated by attention or behavior, which is true for gamma/high-gamma frequencies but not SSVEP. Since the frequencies around the gamma (42-78 Hz) and high-gamma (122-198 Hz) were modulated similarly, frequency smoothening improved discriminability, whereas the frequencies around the SSVEP frequency (20Hz) were not modulated by attention or behavior, and hence frequency smoothening reduced the discriminability for SSVEP. The TW parameter is usually chosen depending on the length of the signal and frequency-band of interest. For relatively wider frequency bands like gamma and high-gamma choosing a time-bandwidth product (TW) of >5 (which smoothens over a frequency bin of >{plus minus} 10 Hz for a 0.5 s signal) is appropriate whereas for narrower bands like alpha (8-12 Hz) and SSVEP the TW is generally kept at a low value. However, when showing power spectral density over a wide range, TW is chosen such that the wider and narrower frequency activities are captured sufficiently well. We have now clarified this in detail in the Results section.

2. In Line 496-519, the results from most panels in Figure 5 are not easy to compare back to Figure 2 and Figure 3 by vague descriptions. They may show similar patterns or trends, but it is still too abstract to be convinced by eyeballing with no statistical testing, e.g. yellow traces in Figure 5A and 3H. If possible, it would be great to show statistical tests and results before making conclusions. It seems to me that these two paragraphs did not provide more information than Figure 6 if there is no statistical tests to support the descriptions. >> Thank you for pointing out this drawback. We agree that the comparison was qualitative in nature. We could not perform statistical analysis such as correlation to measure the similarity between the methods because the binning method had very few frequency points compared to other methods to reliably establish the similarity beyond the visual inspection. Hence, we have now removed the description.

Minor comments:

1. Line 65: typos of citations in the sentence. >> Thank you for noticing. The error has been corrected.

2. Line 282: it may be a typo in "Figure 2-11A-G", because there is no Figure 2-11. >> Thank you for noticing. The typo has been corrected.

3. It would be clearer for readers to have distinct labels for main figures and extended figures. For example, using "Fig. 2E and Extended Fig. 2E" is better than "Fig. 2E and Fig. 2-1E." >> Thank you for the suggestion. We followed this convention to comply with eNeuro's formatting guidelines.

4. Line 790 and 829: the citations are incomplete. >> Thank you for pointing these out. We have corrected the citations.

- Reviewer #2 The paper is written in a complicated manner. The relevance of gamma and high gamma in the behavioural tasks is not clear. The authors have consistently referrred a single paper by Prakash et al for throughout the manuscript, how this study is an advancement in this field and would further help to understand the mechanism of attention and behaviour is lacking. >> Thank you for pointing out this limitation. We have tried to simplify the paper by expanding the acronyms and removing convoluted sentences. We had not made the implications of our study clear earlier so now we have added a paragraph at the end of discussion section where we describe how our study advances the understanding of the mechanisms underlying attention and behavioral outcome in more detail.
