# Supplementary figures and images for: Dissociation of Attentional State and Behavioral Outcome Using Local Field Potentials
Source: eNeuro. 2024 Nov 5;11(11):ENEURO.0327-24.2024. doi: 10.1523/ENEURO.0327-24.2024 (PMC11552547; doi:10.1523/ENEURO.0327-24.2024)

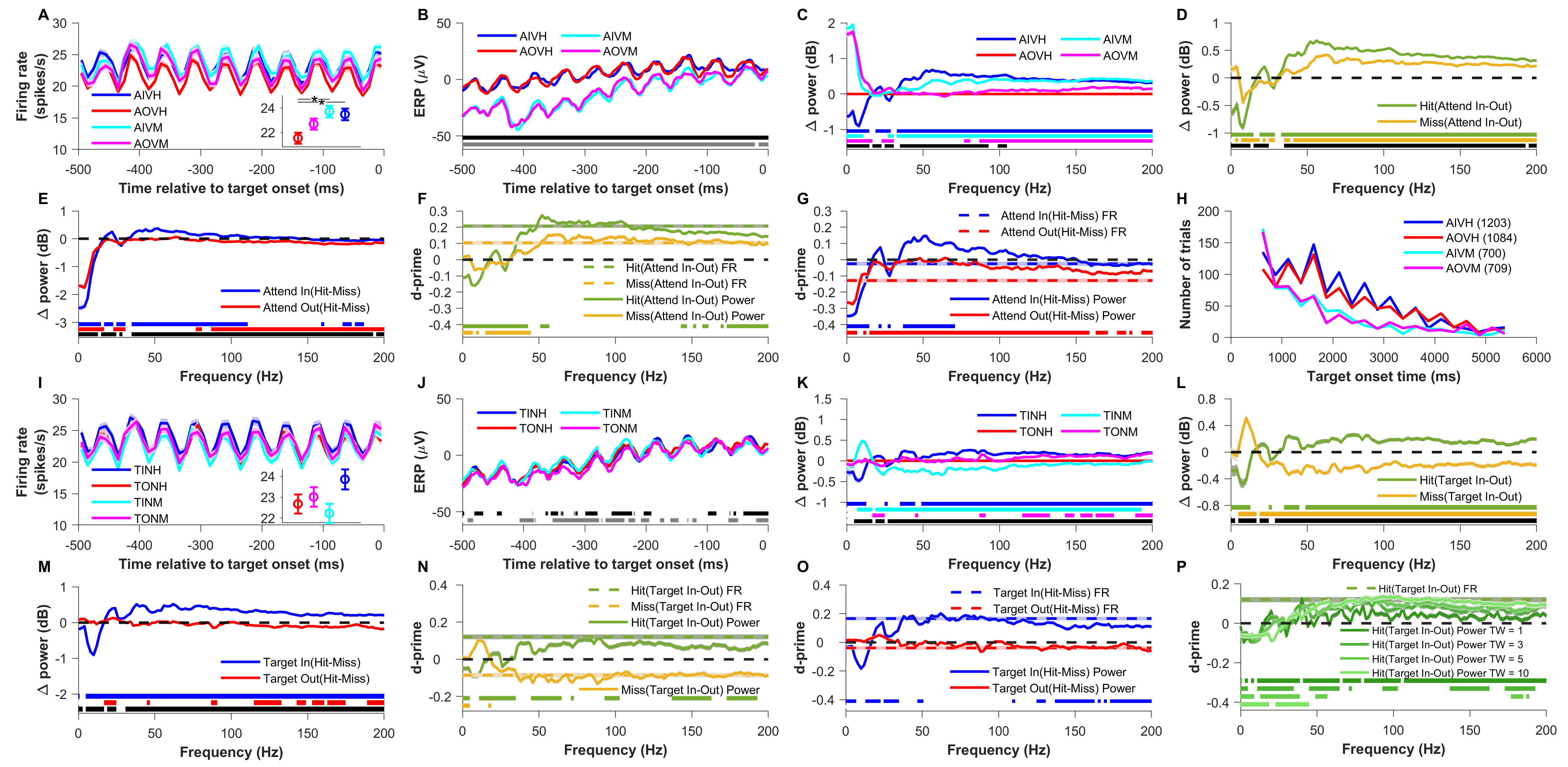

Supplement: Figure 2-1 — Comparison of firing rate (FR) and local field potential (LFP) power across (1) validly cued attention and behavioral conditions for non-matched target onset time distributions and (2) neutrally cued attention and behavioral conditions for matched target onset time distributions. (A) – (G) Same as Figure 2 (A) – (G) but for the case where the target onset time distributions of hit and miss conditions were not matched. Here the mean is taken across 677 electrodes recorded across 22 sessions in two monkeys. Shaded lines and error bars (not visible for most traces) indicate the s.e.m across the 677 electrodes. (H) Frequency distribution of target onset time for the four validly cued conditions of all the sessions. The number in the brackets indicate total number of trials in the respective conditions. (I) Mean peri-stimulus time histogram (PSTH) relative to the target onset time for the neutrally cued conditions in which attention was cued to both visual hemifields simultaneously and target could appear at either of the location with 50% probability. Unlike the valid cue condition where the conditions were divided based on attention location, here the conditions are divided based on where the target eventually appeared, namely Target-In Neutral Hit (TINH; blue), Target-Out Neutral Hit (TONH; red), Target-In Neutral Miss (TINM; cyan), Target-Out Neutral Miss (TONM; magenta). Inset shows the mean firing rate over the same time period as PSTH for the four conditions. Mean is first taken across 659 electrodes recorded across 21 sessions in two monkeys and then averaged across 50 bootstrap iterations. Shaded lines and error bars indicate the bootstrap mean of s.e.m across the 659 electrodes. (J) Mean ERP for the four conditions described in A. (K) Mean change in power spectral density for all the neutrally cued conditions relative to Target-Out Neutral Hit (TONH) condition. (L) Mean change in LFP power spectral density in decibels between Target-In and Target-Out conditions [file eneuro-11-ENEURO.0327-24.2024-s002.tif]

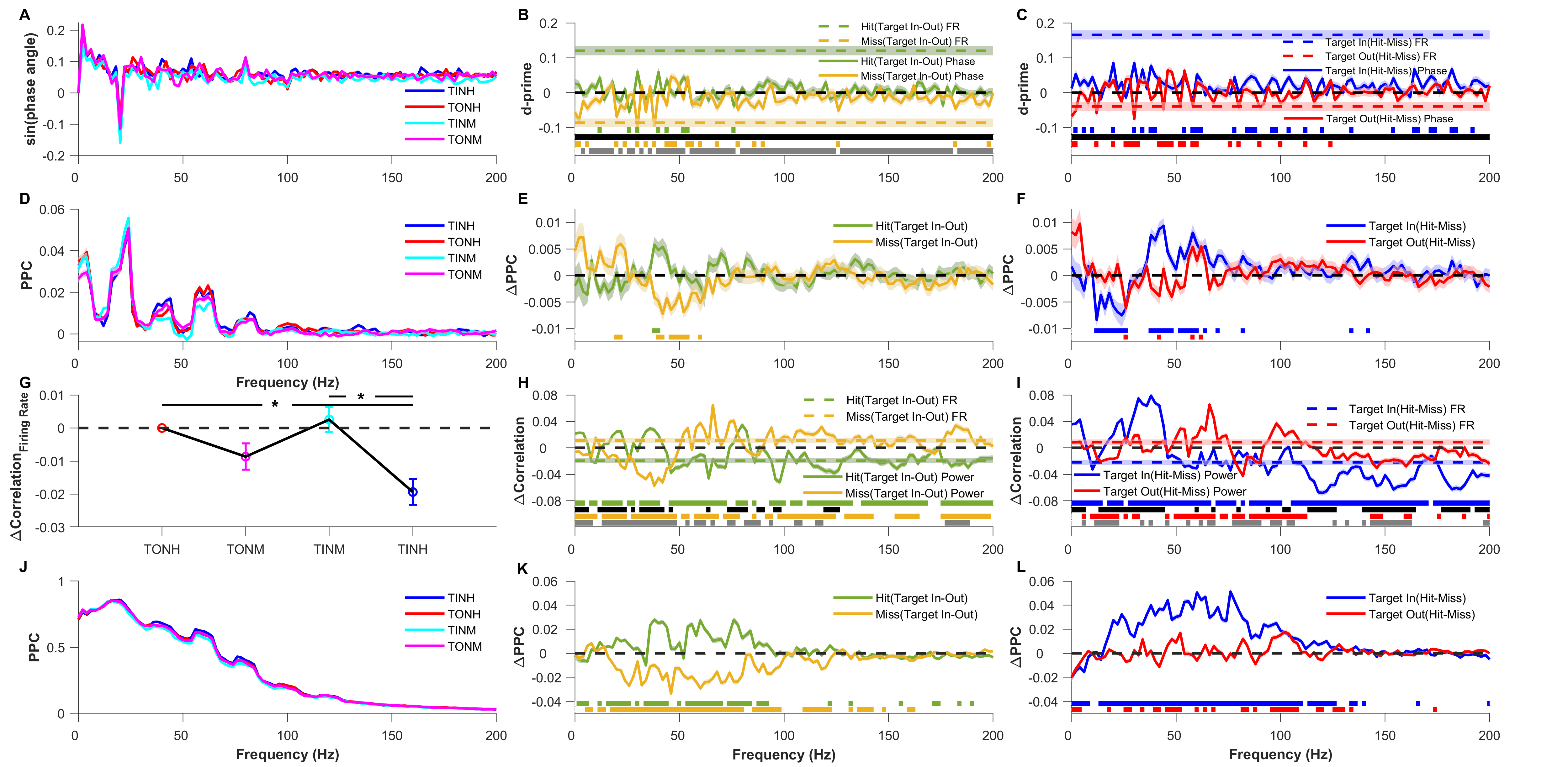

Supplement: Figure 3-1 — Comparison of LFP phase and pairwise phase consistency of individual electrodes and trial-wise firing rate correlation, LFP power correlation and pairwise phase consistency (PPC) of electrode pairs across neutrally cued attention and behavioral conditions. Same as figure 3 but for the neutrally cued conditions. In (A)–(F) mean and s.e.m are computed like in Figure 2-1I – 1O. In (G)-(L) mean was taken across 50 bootstrap samples of mean across 5985 pairs and shaded lines and error bar indicate the bootstrap mean of s.e.m across 5985 electrode pairs. Horizontal color patches at the bottom of each panel indicate the significance level like in Figure 3. Download Figure 3-1, TIF file. [file eneuro-11-ENEURO.0327-24.2024-s003.tif]

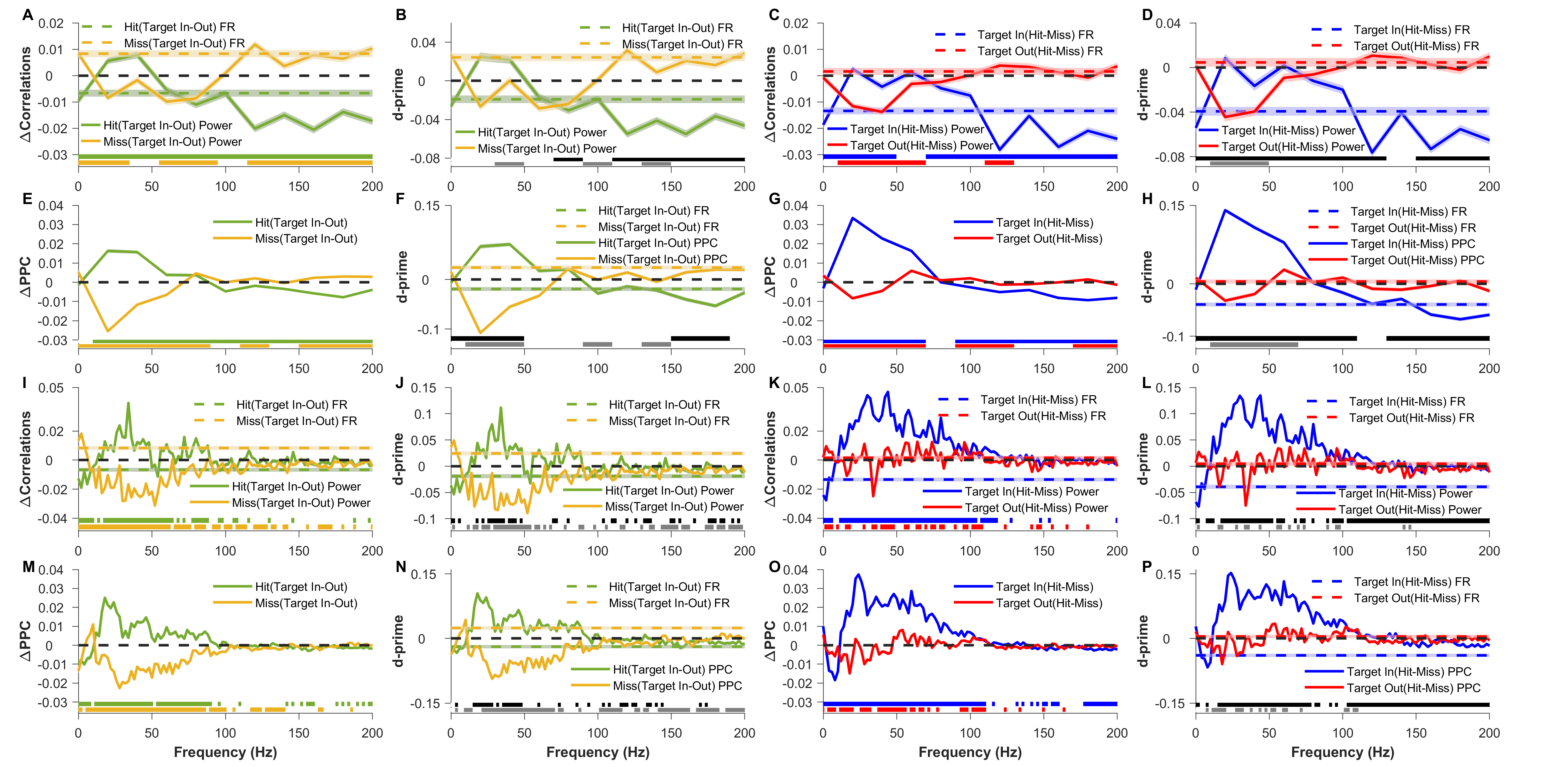

Supplement: Figure 5-1 — Comparison of single trial bin-wise firing rate correlation, bin-wise and taper-wise LFP power correlation and pairwise phase consistency (PPC) across neutrally cued attention and behavioral conditions. Same as figure 5 but for neutrally cued conditions. Mean and s.e.m were computed like in Figure 3-1G – L. Horizontal color patches at the bottom of each panel indicate the significance level like in Figure 5. Download Figure 5-1, TIF file. [file eneuro-11-ENEURO.0327-24.2024-s004.tif]

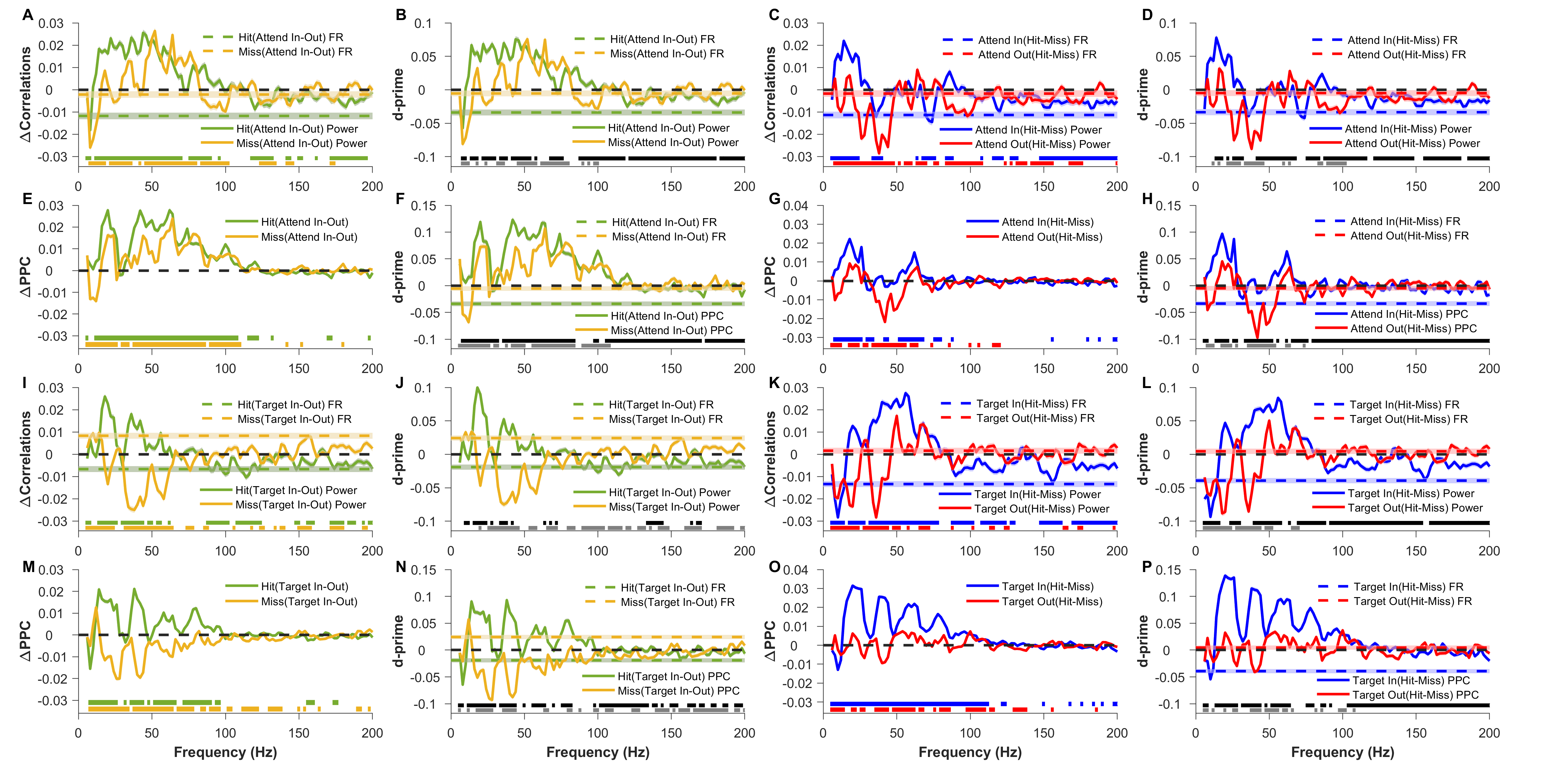

Supplement: Figure 5-2 — Comparison of single trial estimates of LFP power correlation and pairwise phase consistency (PPC) computed using Hilbert transform method with bin-wise firing rate correlation across valid and neutrally cued attention and behavioral conditions. (A)-(H) Same as figure 5I - P. Mean was taken across 50 bootstrap samples of mean across 5754 pairs and shaded lines indicate the bootstrap mean of s.e.m across 5754 electrode pairs. Horizontal color patches at the bottom of each panel indicate the significance level like in Figure 5. (I)-(P) Same as figure 5-1I-P. Mean was taken across 50 bootstrap samples of mean across 5985 pairs and shaded lines indicate the bootstrap mean of s.e.m across 5985 electrode pairs. Horizontal color patches at the bottom of each panel indicate the significance level like in (A)-(H). Download Figure 5-2, TIF file. [file eneuro-11-ENEURO.0327-24.2024-s005.tif]

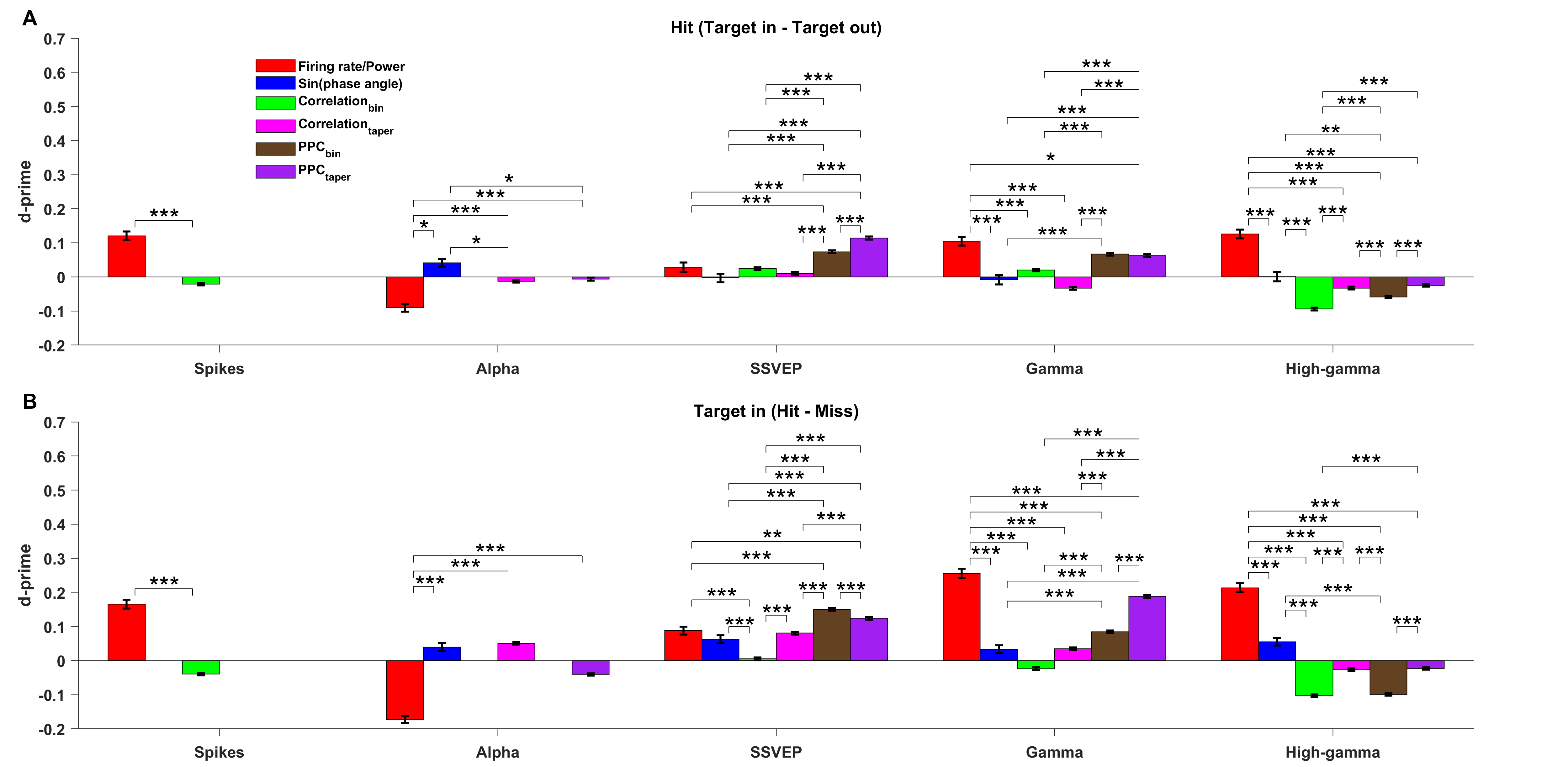

Supplement: Figure 6-1 — Summary plot comparing across the d-primes of all the measures for spikes, LFP power and phase in different frequency bands for neutrally cued attention and behavioral comparison Same as figure 6 but for neutrally cued condition. Asterisks indicate the significance level like in Figure 6. Download Figure 6-1, TIF file. [file eneuro-11-ENEURO.0327-24.2024-s006.tif]
